# Modulation of Gene Expression by Human Cytosolic tRNase Z^L^ through 5′-Half-tRNA

**DOI:** 10.1371/journal.pone.0005908

**Published:** 2009-06-15

**Authors:** Reyad A. Elbarbary, Hiroaki Takaku, Naoto Uchiumi, Hiroko Tamiya, Mayumi Abe, Masayuki Takahashi, Hiroshi Nishida, Masayuki Nashimoto

**Affiliations:** Department of Applied Life Sciences, Niigata University of Pharmacy and Applied Life Sciences, Niigata, Japan; Yale University, United States of America

## Abstract

A long form (tRNase Z^L^) of tRNA 3′ processing endoribonuclease (tRNase Z, or 3′ tRNase) can cleave any target RNA at any desired site under the direction of artificial small guide RNA (sgRNA) that mimics a 5′-half portion of tRNA. Based on this enzymatic property, a gene silencing technology has been developed, in which a specific mRNA level can be downregulated by introducing into cells a synthetic 5′-half-tRNA that is designed to form a pre-tRNA-like complex with a part of the mRNA. Recently 5′-half-tRNA fragments have been reported to exist stably in various types of cells, although little is know about their physiological roles. We were curious to know if endogenous 5′-half-tRNA works as sgRNA for tRNase Z^L^ in the cells. Here we show that human cytosolic tRNase Z^L^ modulates gene expression through 5′-half-tRNA. We found that 5′-half-tRNA^Glu^, which co-immunoprecipitates with tRNase Z^L^, exists predominantly in the cytoplasm, functions as sgRNA *in vitro*, and downregulates the level of a luciferase mRNA containing its target sequence in human kidney 293 cells. We also demonstrated that the PPM1F mRNA is one of the genuine targets of tRNase Z^L^ guided by 5′-half-tRNA^Glu^. Furthermore, the DNA microarray data suggested that tRNase Z^L^ is likely to be involved in the p53 signaling pathway and apoptosis.

## Introduction

**Figure 1:**
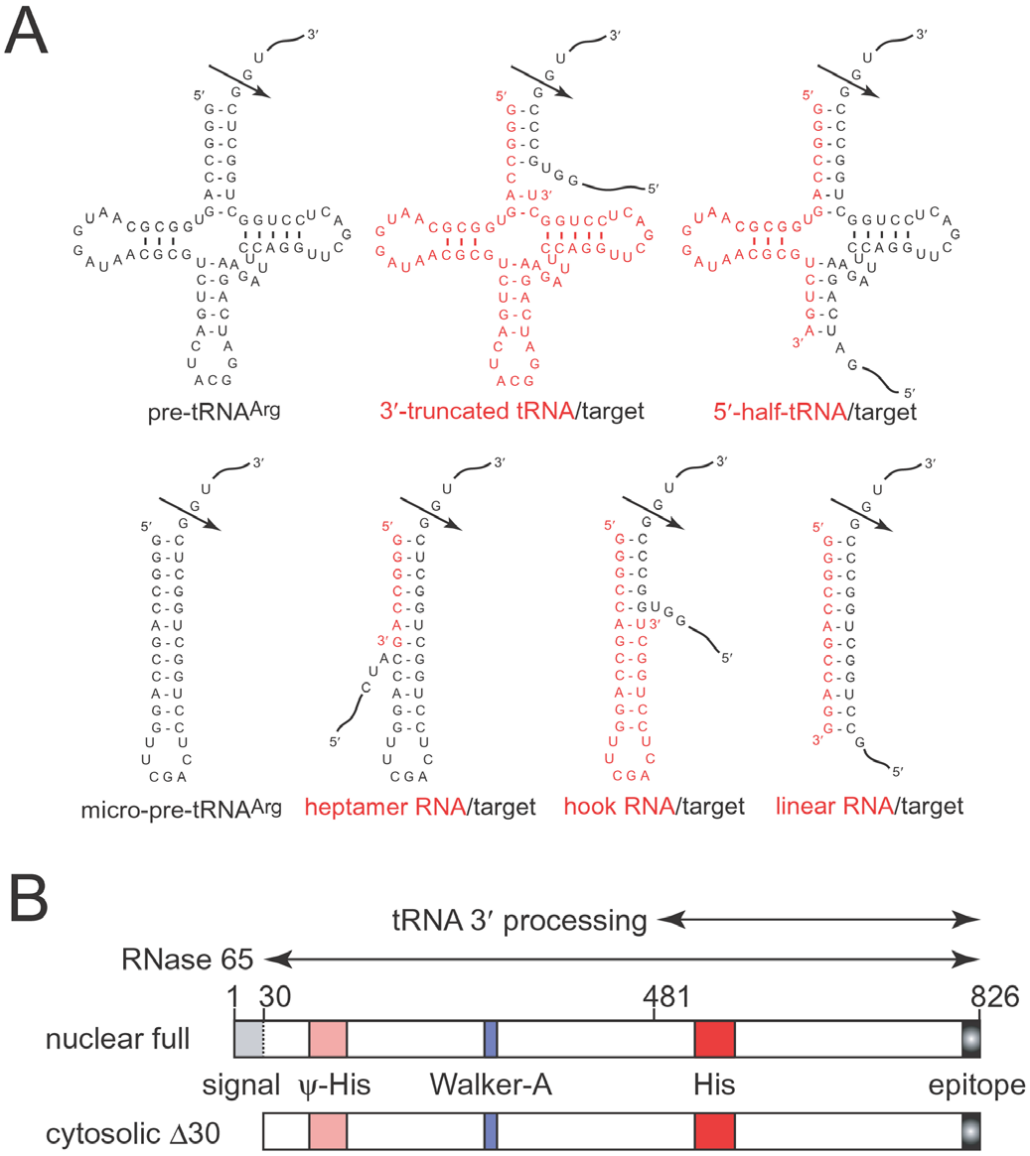
Human tRNase Z^L^ and its substrates. (A) Various RNA substrates for human tRNase Z^L^. Arrows denote the primary cleavage sites. (B) Two forms of human tRNase Z^L^. Double-headed arrows denote minimum regions for the activities [5], [6]. ψ-His, pseudo histidine motif; His, histidine motif.

In 1989, we discovered a unique four-base-recognizing RNA cutter termed RNase 65 in mammalian cytosolic extracts, which is a ribonucleoprotein complex between a long form (tRNase Z^L^) of tRNA 3′ processing endoribonuclease (tRNase Z, or 3′ tRNase) and 3′-truncated tRNA [1]–[3]. We have shown that RNase 65 recognizes substrate RNA via four base-pairings with the 5′-terminal sequence of the 3′-truncated tRNA (Figure 1A), but its role and genuine substrate RNA in the cells remain to be unveiled. tRNase Z is one of the tRNA-maturing enzymes, which removes a 3′ trailer from pre-tRNA [4], [5]. In most cases, tRNase Zs cleave pre-tRNAs immediately downstream of a discriminator nucleotide, onto which the CCA residues are added to produce mature tRNA (Figure 1A). In some cases, additional cleavages occur 1 nt upstream, or 1 or 2 nt downstream. Although mammalian genomes encode an ∼100-kDa long form and an ∼40-kDa short form (tRNase Z^S^), curiously, only tRNase Z^L^ can function as RNase 65 [6].

The finding of RNase 65 encouraged us to pursue further versatility of mammalian tRNase Z^L^, and we have demonstrated *in vitro* that tRNase Z^L^ can cleave any target RNA at any desired site by recognizing a pre-tRNA-like or micro-pre-tRNA-like complex formed between the target RNA and small guide RNA (sgRNA) [7]–[12]. sgRNA is divided into four categories, 5′-half-tRNA, heptamer RNA, hook RNA, and ∼12–16-nt linear RNA (Figure 1A). Again, tRNase Z^S^ can process pre-tRNAs but not the pre-tRNA-like or micro-pre-tRNA-like complexes *in vitro* [6]. We have also shown the efficacy of this RNA targeting method in the living mammalian cells by introducing sgRNAs either as their expression plasmids or as 2′-*O*-methyl RNAs [13]–[16]. We call this method TRUE gene silencing after tRNase Z^L^-utilizing efficacious gene silencing.

A huge number of novel small noncoding RNAs (ncRNAs) are being revealed, which have roles in a great variety of cellular processes [17], [18]. MicroRNAs (miRNAs) are 21–23-nt RNAs that can play important regulatory roles in many processes such as development, cancer, and apoptosis [17]. We have shown that a subset of miRNAs can guide target RNA cleavage by tRNase Z^L^ *in vitro*, and that human miR-103 can downregulate the luciferase gene expression through directing its mRNA cleavage by tRNase Z^L^ [[8, unpublished data]].

Collectively, the cytosolic existence of RNase 65, the efficacy of TRUE gene silencing, and the potential of miRNAs as sgRNAs implied that a new broader gene regulatory system composed of tRNase Z^L^ and small ncRNAs exists in the cells. In order to illuminate this system, we started by trying to catch small RNAs that are interacting with tRNase Z^L^ in the cells. In this study we discovered in human kidney 293 cell extracts various new small ncRNAs including piRNA-like 5′-half-tRNAs and 28S rRNA fragments, co-immunoprecipitated with tRNase Z^L^, and demonstrated that these ncRNAs work as sgRNAs for tRNase Z^L^ *in vivo* as well as *in vitro*. Further, we confirmed that tRNase Z^L^ exists ubiquitously in a human cell. Taken together, the present data suggest that human cytosolic tRNase Z^L^ modulates gene expression through various types of small ncRNA. We also show that PPM1F and DYNC1H1 mRNAs are its genuine targets, and discuss its physiological roles.

## Results

### piRNA-like ncRNAs co-immunoprecipitate with human tRNase Z^L^

Since we were curious to know if other types of cellular small ncRNAs could work as sgRNAs for tRNase Z^L^, we tried to catch small RNAs that are interacting with tRNase Z^L^ in the cells by isolating RNAs from 293 cell extracts which co-immunoprecipitate with tRNase Z^L^. We cloned and sequenced 26 RNAs of ∼20–40 nt, and identified 11 5′-half or 3′-half tRNA fragments, 6 rRNA fragments, 4 snRNA fragments, one fragment of the DOCK9 pre-mRNA, and 4 RNA fragments of unknown origin (Table S1). To our surprise, 6 tRNA fragments and all of the rRNA fragments were very closely related to piRNAs, which have been found in germ line cells [18]. One of the snRNA fragments, the U6B snRNA fragment, also matched with an mRNA for a β-actin-like protein. Some of these RNAs may be background noise since we also identified one 18S rRNA fragment in a negative control. To confirm that these tRNase Z^L^-interacting small ncRNAs really exist in the cells, we performed northern analysis for the several representative ncRNAs. Although the expression levels differed with the species, most of the ncRNAs were detected in both 293 and HeLa cells, and the 5′-half-tRNA^Glu^ was predominant in the cytoplasm in the 293 cells (Figure S1). Recently, other groups have also reported that 5′-half and 3′-half tRNA fragments exist stably in various types of cells, although little is know about their physiological roles [19]–[23].

### 5′-half-tRNA^Glu^ functions as sgRNA *in vitro*

**Figure 2:**
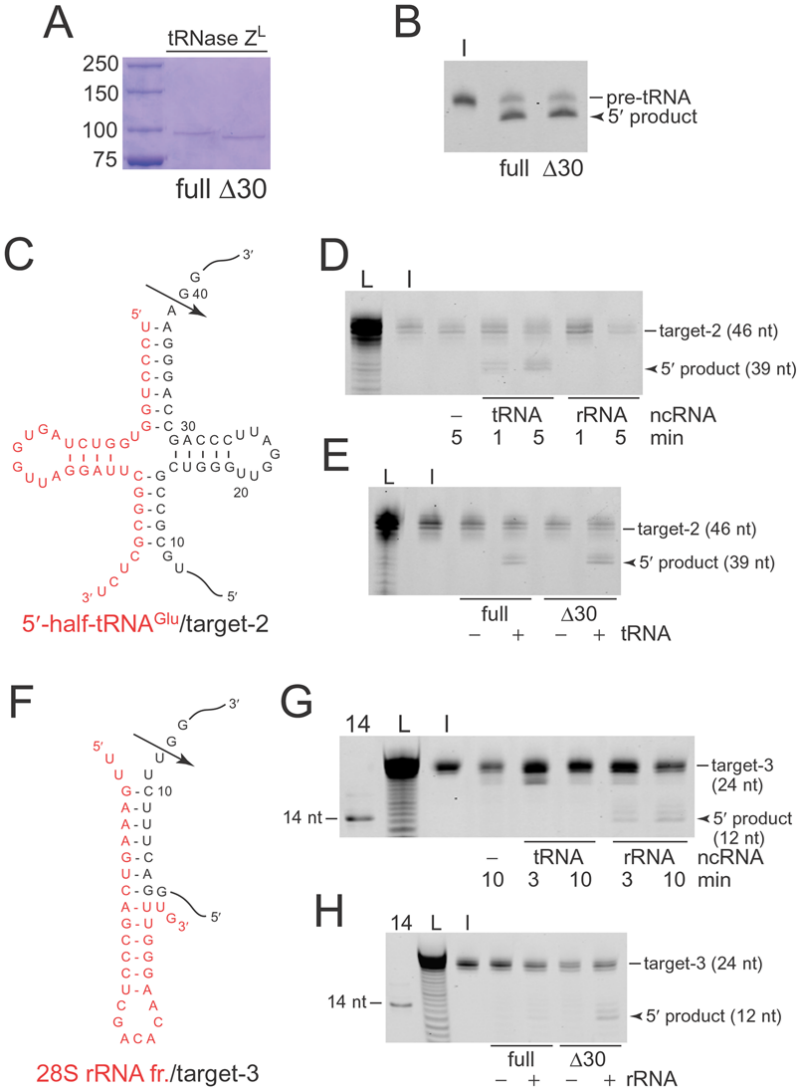
A 5′-half-tRNA^Glu^ and a 28S rRNA fragment function as sgRNAs *in vitro*. (A) Purified histidine-tagged full-length and Δ30 tRNase Z^L^ proteins separated on an SDS/polyacrylamide gel. (B) 5′-fluorescein-labeled pre-tRNA^Arg^ was reacted with the full-length and Δ30 enzymes for 5 min, and the products were analyzed on a denaturing polyacrylamide gel. I, input. (C) A secondary structure of the 5′-half-tRNA^Glu^/target-2 RNA complex. An arrow denotes the primary cleavage site. (D) 5′-fluorescein-labeled target-2 was incubated with recombinant human Δ30 tRNase Z^L^ in the absence or presence of the 5′-half-tRNA^Glu^ or the 28S rRNA fragment. The cleavage products were analyzed on a denaturing polyacrylamide gel. L, alkaline ladder of the target-2; I, input. (E) The 5′-half-tRNA^Glu^/target-2 complex was also reacted with the full-length and Δ30 enzymes for 5 min. (F) A secondary structure of the 28S rRNA fragment/target-3 RNA complex. An arrow denotes the primary cleavage site. (G) 5′-fluorescein-labeled target-3 was incubated with recombinant human Δ30 tRNase Z^L^ in the absence or presence of the 5′-half-tRNA^Glu^ or the 28S rRNA fragment. The cleavage products were analyzed on a denaturing polyacrylamide gel. 14, 14-nt size standard; L, alkaline ladder; I, input. (H) The cleavage reactions were also performed with the full-length and Δ30 enzymes for 5 min.

The two 5′-half-tRNAs among the tRNase Z^L^-co-immunoprecipitated ncRNAs especially attracted us to further investigation since these 5′-half-tRNAs are the very RNAs that we have been using as tailor-made sgRNAs for specific gene silencing by tRNase Z^L^. We examined a 5′-half-tRNA^Glu^ for its capability for sgRNA *in vitro*. A target RNA, target-2, was designed to include a sequence partially complementary to each RNA and to form a pre-tRNA-like structure, when bound (Figure 2C). As we show below, there exist two forms of human tRNase Z^L^, full-length and N-terminal-truncated ones (Figures 1B and 2A). We first tested Δ30 tRNase Z^L^ lacking the N-terminal 30 amino acids, which are predicted to correspond to the mitochondrial transport signal [24]. The target-2 was efficiently cleaved at the expected 39th-nt site in the presence of the 5′-half-tRNA^Glu^ but not in the presence of the 30-nt 28S rRNA fragment that was co-immunoprecipitated with tRNase Z^L^ (Figure 2D). We also tested full-length tRNase Z^L^ for cleavage of the complex. The full-length enzyme was slightly less active than the Δ30 one (Figure 2E), whereas both enzymes cleaved pre-tRNA^Arg^ with the same efficiency (Figure 2B).

We also found that the 28S rRNA fragment can form a hook structure and thus can potentially work as sgRNA (Figures 1A and 2F) [9]. To examine if this is the case, we designed the RNA target-3 to form a micro-pre-tRNA-like structure with the 28S rRNA fragment (Figure 2F). The target-3 was cleaved at the expected 12th-nt site only in the presence of the 28S rRNA fragment (Figure 2G), and in this case, the Δ30 tRNase Z^L^ was much more active than the full-length enzyme (Figure 2H), indicating that the 28S rRNA fragment indeed works as hook-type sgRNA.

### Human tRNase Z^L^ exists ubiquitously in cells

**Figure 3:**
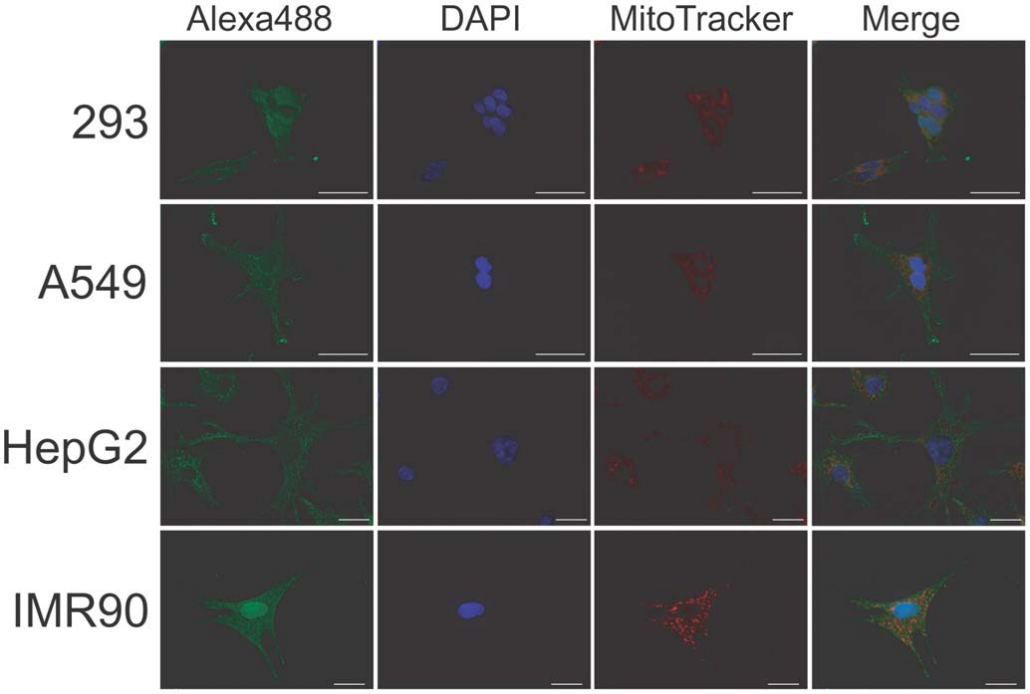
Human tRNase Z^L^ exists ubiquitously in cells. Fluorescent microscopic analyses of human 293 kidney cells, A549 epithelial lung cells, HepG2 hepatoma cells, and IMR90 lung fibroblasts. The cells were incubated with primary tRNase Z^L^ antibodies and subsequently with an Alexa488-conjugated secondary antibody (Alexa488). Negative control pictures are shown in Figure S2. DAPI was used to stain DNA (DAPI), and MitoTracker Red was to stain the mitochondria (MitoTracker). Bar, 20 µm.

If tRNase Z^L^ really works with 5′-half-tRNA^Glu^, which is predominant in the cytoplasm, it would need to exist in the cytosol as well as in the nuclei and the mitochondria, where the tRNA 3′ processing occurs. And the above *in vitro* observation implies that the cytosolic form should be the Δ30 tRNase Z^L^ rather than the full-length one. We examined the intracellular location of tRNase Z^L^ by the indirect fluorescent method using polyclonal antibodies against a human tRNase Z^L^ peptide (C-terminal amino acid 812–826). Fluorescent microscopic analysis showed that tRNase Z^L^ exists everywhere in the 293 cells although the amount of the nuclear tRNase Z^L^ appears to vary depending on the cells (Figures 3 and S2). The similar distribution patterns were observed in human A549 epithelial lung adenocarcinoma cells, HepG2 hepatoma cells, and IMR90 lung fibroblasts (Figure 3 and S2), suggesting that tRNase Z^L^ is ubiquitous in any type of human cells.

### Δ30 tRNase Z^L^ exists primarily in the cytosol

**Figure 4:**
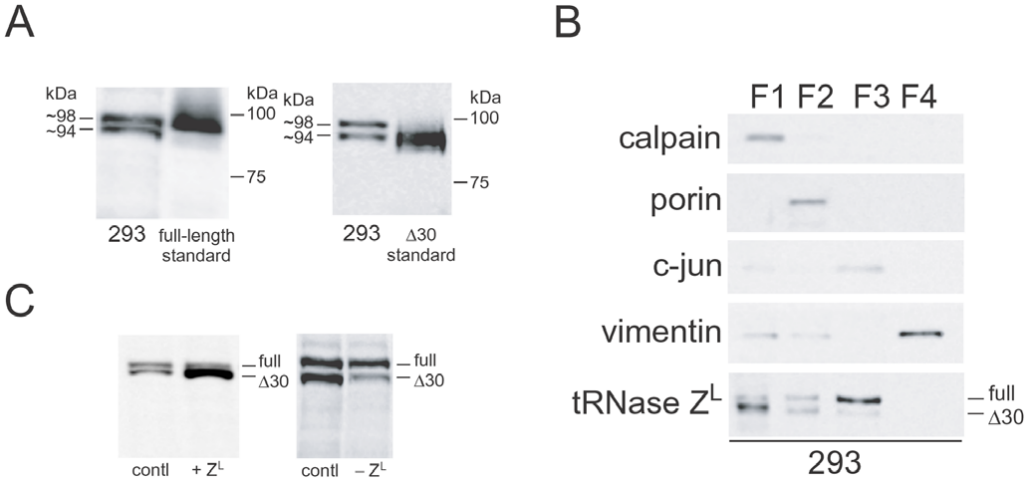
Δ30 tRNase Z^L^ exists primarily in the cytosol. (A) Western blotting for human tRNase Z^L^ from the 293 cells. The recombinant full-length and Δ30 tRNase Z^L^ standards were generated from intein-fusion proteins. The membrane was probed with antibodies raised to a human tRNase Z^L^ peptide (amino acid 812–826). (B) Western analyses for human tRNase Z^L^ in the subcellular fractions. The cytosolic (F1), membrane/organelle (F2), nuclear (F3), and cytoskeletal (F4) fractions were prepared from the 293 cells. (C) Total protein samples from the 293 cells that were transfected with the tRNase Z^L^ expression plasmid (+Z^L^) or with the tRNase Z^L^ siRNA (−Z^L^) were subjected to western analyses.

Western blotting showed that in the 293 cells exist ∼98- and ∼94-kDa forms of tRNase Z^L^, which correspond to the full-length and Δ30 enzymes, respectively (Figure 4A). To elucidate a subcellular distribution of these two forms, we carried out the western analysis for tRNase Z^L^ in subcellular fractions, and found that, as expected, the Δ30 form exists primarily in the cytosolic fraction while the full-length form is primarily in the nuclear fraction (Figure 4B). Both forms were also found in the membrane/organelle fraction.

Introduction of the tRNase Z^L^ expression plasmid or the tRNase Z^L^ siRNA into the 293 cells changed the level of the Δ30 form more drastically than that of the full-length one (Figure 4C). This observation suggests that there may be a mechanism to maintain the full-length tRNase Z^L^ level in the nuclei and that the role of the nuclear tRNase Z^L^ may be more important than that of the cytosolic Δ30 tRNase Z^L^.

### 5′-half-tRNA^Glu^ works as sgRNA *in vivo*

**Figure 5:**
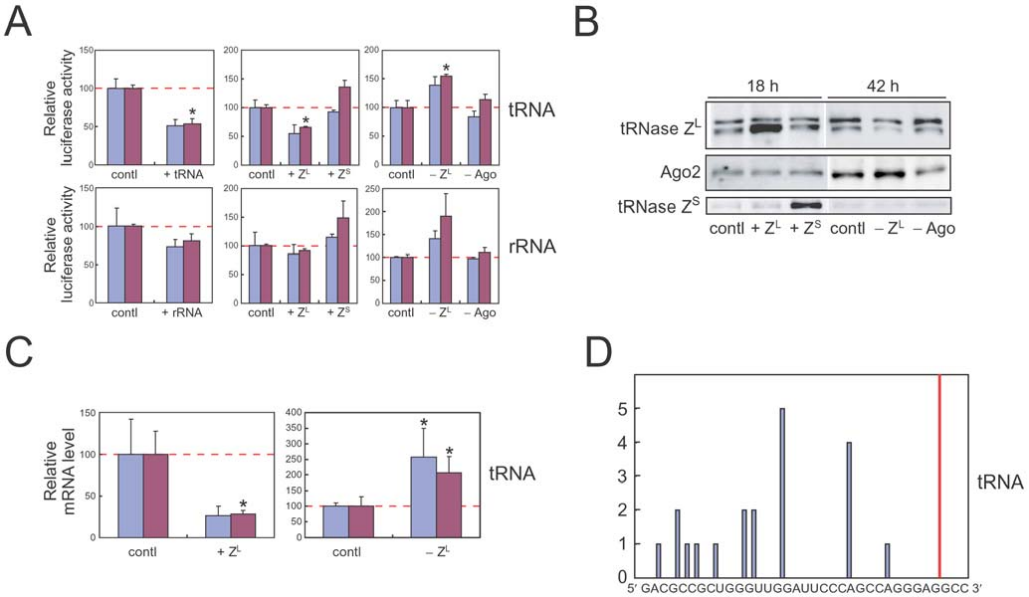
5′-half-tRNA^Glu^ works as sgRNA *in vivo*. (A) Luciferase assays with the 293 cells. The ratio of the expression level from mlucT(tRNA) or mlucT(rRNA) to that from the unmodified luciferase mRNA mlucT_0_ is shown in the 293 cells that were co-transfected with 5′-half-tRNA^Glu^ (+tRNA), the 28S rRNA fragment (+rRNA), the tRNase Z^L^ expression plasmid (+Z^L^), the tRNase Z^S^ expression plasmid (+Z^S^), the tRNase Z^L^ siRNA (−Z^L^), or the Ago2 siRNAs (−Ago). Two independent sets of data are presented, and error bars indicate s.d. (n = 3). Asterisk, *P*<0.01. (B) Western blotting for tRNase Z^L^, Ago2, and tRNase Z^S^ in the 293 cells that were transfected with the above plasmids or siRNAs. (C) The ratio of the mlucT(tRNA) amount to the mlucT_0_ amount in the 293 cells quantitated by real-time PCR. Two independent sets of data are presented, and error bars indicate s.d. (n = 3). Asterisk, *P*<0.05. (D) Distribution of 3′ ends of 5′ cleavage products of mlucT(tRNA). Red line, expected cleavage site.

Next, we performed *in vivo* analysis using a luciferase reporter plasmid modified to contain the target sequence in the 3′ UTR. The expression level from the luciferase mRNA mlucT(tRNA) containing the 5′-half-tRNA^Glu^ target sequence was decreased by adding the synthetic 5′-half-tRNA^Glu^ into the 293 cells compared with that from the normal luciferase mRNA mlucT_0_ (Figure 5A). The downregulation level was augmented by over-expressing tRNase Z^L^ and lowered by decreasing the tRNase Z^L^ level (Figure 5A,B). In contrast, the over-expression of tRNase Z^S^ slightly decreased the downregulation level, and the Ago2 downregulation did not affect the expression significantly. The same effect of the change in the tRNase Z^L^ level on the mlucT(tRNA) amount itself was also observed (Figure 5C). We also analyzed 3′-end sequences of 5′ cleavage products from mlucT(tRNA), and identified the 3′ ends at 6- to 30-nt upstream of the expected tRNase Z^L^ cleavage site (Figure 5D). The shorter cleavage products could be due to 3′ to 5′ exoribonuclease reactions after the tRNase Z^L^ cleavage, although there is no direct evidence yet. In contrast, we could not clone any fragments from the unmodified luciferase mRNA, supporting the involvement of the 5′-half-tRNA^Glu^ as sgRNA in the cleavage of mlucT(tRNA). In the similar fashion, the 28S rRNA fragment and tRNase Z^L^ affected the expression from mlucT(rRNA) containing the target sequence of the 28S rRNA fragment (Figure 5A).

### The PPM1F mRNA is a genuine target of tRNase Z^L^ guided by 5′-half-tRNA^Glu^

Taken together, these results imply that tRNase Z^L^ together with naturally occurring small ncRNAs plays a role in regulation of gene expression. In order to find genuine mRNA targets of tRNase Z^L^ guided by ncRNAs, we performed DNA microarray analysis for mRNAs from the 293 cells transfected with the tRNase Z^L^ expression plasmid. The expressions of 41 and 310 genes were downregulated and upregulated by >1.5-fold, respectively, in the presence of a higher level of tRNase Z^L^. It is likely that a subset of the 41 downregulated mRNAs are direct targets of tRNase Z^L^ (Figure S3A). The Kyoto Encyclopedia of Genes and Genomes (KEGG) pathway analysis (http://www.genome.ad.jp/kegg) [25] suggested that tRNase Z^L^ is involved in the p53 signaling pathway and apoptosis with P values = 1.3×10^−6^ and = 4.2×10^−4^, respectively (Figure S3B,C). We also searched a human mRNA database (http://www.ncbi.nlm.nih.gov) for potential mRNA targets of tRNase Z^L^ guided by 5′-half-tRNA^Glu^, and retrieved 432 mRNAs, which contain one to nine potential binding sites for 5′-half-tRNA^Glu^ (Table S2). The criteria of its binding site are the presence of 7-nt downstream and 3- to 5-nt upstream sequences that are complementary to the acceptor-stem and anticodon-stem domain sequences, respectively, of the 5′-half-tRNA^Glu^, and a T-arm-like structure in between. DYNC1H1, LYPLA2, MXD4, PBX2, PPM1F, RBM6, SREBF2, and ZNF609 mRNAs were found in both groups (Figure S3A and Table S2).

To identify specific mRNA targets of tRNase Z^L^ directed by 5′-half-tRNA^Glu^, we arbitrarily selected DYNC1H1, KIF1A, LYPLA2, PPM1F, RBM6, SREBF2, ZFAND6, ZNF609, and ZDHHC20 mRNAs from the mRNAs downregulated in the presence of a higher level of tRNase Z^L^ (Figure S3A), and CTDSP2, FOXP2, and HDAC4 mRNAs from the potential 5′-half-tRNA^Glu^ target mRNAs (Table S2), and analyzed changes in their amounts in response to the change in the intracellular level of the 28S rRNA fragment, the 5′-half-tRNA^Glu^, or tRNase Z^L^. First, we performed non-quantitative reverse-transcription PCR analysis to see the changes in mRNA levels using the 28S rRNA level as an internal standard, and observed obvious changes in the PPM1F, DYNC1H1, and HDAC4 mRNA levels (Figure S4). These mRNAs contain 0, 6, and 9 potential 28S rRNA fragment binding sites, and 3, 1, and 2 potential 5′-half-tRNA^Glu^ binding sites, respectively. Possible secondary structures of 5′-half-tRNA^Glu^/PPM1F mRNA complexes are exampled in Figure S5. There were no obvious changes in the levels of the other mRNAs such as FOXP2 mRNA (Figure S4 and data not shown). The PPM1F mRNA level was decreased by increasing the level of 5′-half-tRNA^Glu^ or tRNase Z^L^, but not affected by increasing the 28S rRNA fragment level, whereas the DYNC1H1 mRNA level was decreased by increasing the level of the 28S rRNA fragment or tRNase Z^L^, but not affected by increasing the 5′-half-tRNA^Glu^ level. Curiously, the HDAC4 mRNA level increased with the increase in the level of 5′-half-tRNA^Glu^ or tRNase Z^L^, but did not change by increasing the 28S rRNA fragment level. On the whole, multiple target sites in an mRNA for an ncRNA appear to be essential for efficient downregulation, although obviously this rule does not hold in the HDAC4 case.

**Figure 6:**
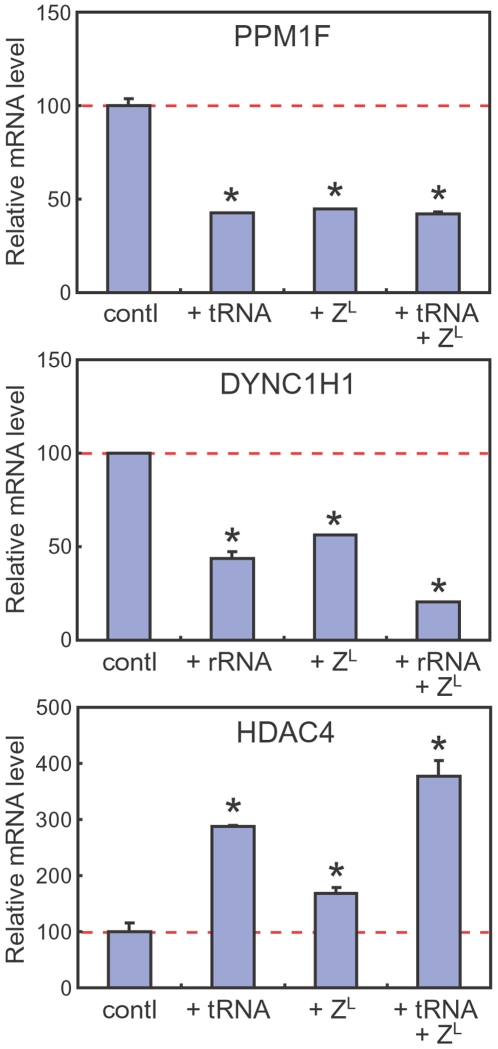
PPM1F and DYNC1H1 mRNAs are targets of ncRNA-guided tRNase Z^L^. Total RNA was extracted from the 293 cells that were transfected with 5′-half-tRNA^Glu^ (+tRNA) or the 28S rRNA fragment (+rRNA) and/or the tRNase Z^L^ expression plasmid (+Z^L^), and analyzed by real-time PCR. The mRNA levels are normalized against the 28S rRNA levels. Error bars indicate s.d. (n = 3). Asterisk, *P*<0.005.

To confirm and quantitate the changes in the mRNA levels, we carried out real-time PCR using different pairs of primers. Again, the PPM1F mRNA level was downregulated up to less than 50% by increasing the level of 5′-half-tRNA^Glu^ or tRNase Z^L^, and the DYNC1H1 mRNA level was downregulated up to ∼50% by increasing the level of the 28S rRNA fragment or tRNase Z^L^ (Figure 6). The DYNC1H1 mRNA level decreased further up to ∼20% by increasing both levels of the 28S rRNA fragment and tRNase Z^L^, while the decrease in the PPM1F mRNA level was hardly affected by increasing both levels of 5′-half-tRNA^Glu^ and tRNase Z^L^, suggesting that about half of the PPM1F mRNA molecules may be protected from degradation by somehow compartmentalizing them. The HDAC4 mRNA level upregulated up to 2.9-, 1.7-, and 3.8-fold by increasing the levels of 5′-half-tRNA^Glu^, tRNase Z^L^, and both, respectively (Figure 6).

We also analyzed 3′-end sequences of 5′ cleavage products from the PPM1F mRNA, and identified the 3′ ends at 6- and 43-nt upstream of the first site of the three expected tRNase Z^L^ cleavage sites (Figures S5 and S6). The shorter cleavage products could have been caused by 3′ to 5′ exoribonuclease reactions after the tRNase Z^L^ cleavage. No PPM1F mRNA cleavage products were detected at the other two sites.

These results suggest that the PPM1F and DYNC1H1 mRNAs are genuine targets of tRNase Z^L^ guided by 5′-half-tRNA^Glu^ and the 28S rRNA fragment, respectively. In support of this, one out of three potential 5′-half-tRNA^Glu^-guided tRNase Z^L^ target sequences in the human PPM1F mRNA and all six potential 28S-rRNA-fragment-guided tRNase Z^L^ target sequences in the human DYNC1H1 mRNA are well conserved among human, mouse, and rat at most with single base-pair-disrupting nt substitutions in the ncRNA-binding sites (Figure S7). The mouse and rat sequences corresponding to the downstream 5′-half-tRNA^Glu^-binding sequence in the second target site of the human PPM1F mRNA contain triple base-pair-disrupting nt substitutions (Figure S7A), and mouse and rat sequences corresponding to the third target sequence in the 3′ UTR of the human PPM1F mRNA are not discernible. Together with the observation that the human PPM1F mRNA fragments were detected only at the first target site (Figure S6), these suggest that the second and third target sites might not be used in human cells also. The unexpected changes in the HDAC4 mRNA level would be explained by assuming that the HDAC4 mRNA level is downregulated by proteins from some mRNAs and/or some ncRNAs, the levels of which are downregulated by tRNase Z^L^ guided by 5′-half-tRNA^Glu^.

## Discussion

### Origin of tRNase Z^L^-interacting ncRNA

We identified 26 small RNA species of ∼20–40 nt that co-immunoprecipitated with human tRNase Z^L^. These RNAs are 11 tRNA fragments, 6 rRNA fragments, 4 snRNA fragments, one fragment of the DOCK9 pre-mRNA, and 4 RNA fragments of unknown origin (Table S1). The existence of some of these RNAs in the cells was shown by northern analysis (Figure S1). We believe that the fragments of tRNAs, rRNAs, and snRNAs would function as sgRNAs and that the 4 RNA fragments of unknown origin may be parts of target mRNAs to be cleaved by cytosolic Δ30 tRNase Z^L^ under the direction of cellular ncRNAs functioning as sgRNAs.

If the 21-nt RNA that matches with the U6B snRNA fragment turns out to be from the mRNA for the β-actin-like protein, this RNA may be also a target of cytosolic Δ30 tRNase Z^L^. The fragment of the DOCK9 pre-mRNA is from one of its introns, suggesting that the DOCK9 expression may be modulated by nuclear tRNase Z^L^ under the direction of nuclear small ncRNA and/or that this RNA fragment, which is generated from the intron through an as yet unknown mechanism, may work as sgRNA. Although we showed that the 5′-half-tRNA^Glu^ and the 28S rRNA fragment work as 5′-half-tRNA-type and hook-type sgRNAs, respectively, we do not necessarily know the guiding modes of the other newly identified ncRNAs. It is also possible that 3′-half-tRNAs function as 5′-half-tRNA-type sgRNA.

**Figure 7:**
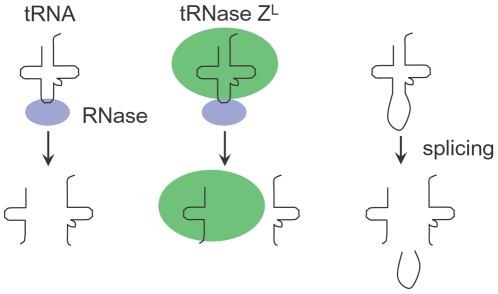
Mechanisms for generation of half-tRNAs as sgRNAs. See text for a detailed description. Possible pathways for generation of half-tRNA fragments that can be used as sgRNAs.

Although we do not know currently how these potential sgRNAs are generated from tRNAs, rRNAs, and snRNAs, we would like to propose possible pathways for generation of half-tRNA fragments, which are not mutually exclusive (Figure 7). A free tRNA molecule may be cleaved in the anticodon loop by some specific endoribonuclease(s) to produce 5′-half-tRNA and 3′-half-tRNA molecules. This tRNA molecule to be processed may be a functional one or a non-functional misfolded one. Alternatively, a tRNA molecule bound to tRNase Z^L^ may be processed likewise, because the acceptor stem and the T arm are the minimum requirements for tRNase Z^L^ recognition and the anticodon loop would be accessible to another enzyme. In this case, the 3′-half-tRNA molecule may be discarded and the 5′-half-tRNA molecule may be used directly as sgRNA inside tRNase Z^L^. The discarded 3′-half-tRNA molecule may be also used as 5′-half-tRNA-type sgRNA by another tRNase Z^L^ molecule. Recently, the RNase T2 family member Rny1 and the RNase A family member angiogenin have been shown to be responsible for the half-tRNA generation in yeast and human cells, respectively [22], [23]. Another possibility is that the half-tRNA molecules may be generated as splicing intermediate molecules from intron-containing tRNAs. In any case, tRNA sources would be from genuine gene transcripts and/or from pseudogene transcripts, because the highly conserved A_58_ was substituted with U_58_ in six cases and with C_58_ in one case among the nine 3′-half-tRNAs (Table S1).

### The Δ30 form of human tRNase Z^L^

We identified two forms of human tRNase Z^L^: one is the nuclear full-length form and the other is the cytosolic Δ30 form (Figure 4B). Both forms are also found in the membrane/organelle fraction. Since the N-terminal 30 amino acids are predicted to be a mitochondrial transport signal [24], probably the full-length form would be one that is bound to the mitochondrial membrane and is waiting for processing, whereas the Δ30 form would be a processed one inside the mitochondria. The nuclear transport of the full-length tRNase Z^L^ would be accomplished through a potential nuclear localization signal, _28_RRERPRKD_35_, at the N-terminal region [26]. We, however, do not know a mechanism for generation of the Δ30 form in the cytosol. The mitochondrial Δ30 enzyme may be retrograde-transported to the cytosol, or some specific processing enzyme in the cytosol may be responsible for its production. In any case, once the Δ30 form is generated in the cytosol, it would be hardly transported to the nucleus, because the Δ30 enzyme lacks the first three amino acids of the potential nuclear localization signal. This is consistent with the observation that the Δ30 form was barely detected in the nuclear fraction (Figure 4B).

Curiously, the Δ30 tRNase Z^L^ was much more active than the full-length enzyme on the 28S rRNA fragment/target-3 complex, whereas the activities of both enzymes were the same on the pre-tRNA^Arg^ substrate (Figures 2B,H). With respect to the 5′-half-tRNA^Glu^/target-2 complex, the Δ30 form was slightly more active than the full-length one (Figure 2E). These observations imply that the N-terminal 30 amino acids may somehow inhibit the recognition of micro-pre-tRNA-like complexes by tRNase Z^L^. To elucidate the structural basis for this issue, we are currently trying to solve crystal structures of both forms with RNA substrates.

### Proteins that interact with human tRNase Z^L^

Because human tRNase Z^L^ exists everywhere in the cells (Figure 3) and is a relatively large enzyme, it would be expected to interact physically with other proteins. Indeed, human tRNase Z^L^ has been shown to interact with γ-tubulin and two other protein components of the γ-tubulin ring complex by co-immunoprecipitation experiments [27]. The interaction between tRNase Z^L^ and α-tubulin, however, has not been observed, suggesting that human tRNase Z^L^ interacts with a free form of the γ-tubulin ring complex. By co-immunoprecipitation experiments with the tRNase Z^L^ antibodies, we confirmed the tRNase Z^L^/γ-tubulin interaction and found that tRNase Z^L^ also interacts with myosin II under some conditions (data not shown). Consistent with this, Δ30 tRNase Z^L^ was detected in the cytoskeletal fraction by the western analysis for tRNase Z^L^ in subcellular fractions from the 293 cells transfected with the tRNase Z^L^ expression plasmid (data not shown).

Human tRNase Z^L^ was also reported to interact with Smad2 to function as a transcription factor by co-immunoprecipitation experiments using exogenously expressed tagged human tRNase Z^L^ [28]. Although this observation is potentially very intriguing, we could not detect this interaction with respect to endogenous human tRNase Z^L^.

### Genuine targets and physiological roles

We found that the DYNC1H1 mRNA is likely to be a genuine target of tRNase Z^L^ (Figure 6), and the two RNA fragments co-immunoprecipitated with tRNase Z^L^ suggest that the β-actin-like protein mRNA and the DOCK9 pre-mRNA may also be genuine targets (Table S1). The dynein's function and putative functions of the β-actin-like protein and DOCK9 [29], [30] together with the presence of the γ-tubulin/tRNase Z^L^ and myosin II/tRNase Z^L^ interactions [27] (data not shown) appear to converge at the supposition that tRNase Z^L^ is involved in regulation of mitosis.

The DNA microarray data suggested that tRNase Z^L^ is likely to be involved in the p53 signaling pathway and apoptosis (Figure S3B,C), and PPM1F, the mRNA of which was identified as a target of tRNase Z^L^ guided by 5′-half-tRNA^Glu^ (Figure 6), has been shown to induce apoptosis in HeLa cells when overexpressed [31]. Interestingly, calcium/calmodulin-dependent protein kinase II, which is regulated by PPM1F in fibroblasts [32], phosphorylates the product of the HDAC4 mRNA [33], the level of which was upregulated by tRNase Z^L^ and 5′-half-tRNA^Glu^ (Figure 6). Furthermore, the observations that the 5′-half-tRNA^Glu^ level in both 293 and HeLa cells increases with the increase in cell density (Figure S8A) and that the level of the RNase 65 RNA component 3′-truncated tRNA^Arg^ increases in BJAB cells after adding 5-fluorouracil (Figure S8B) suggest their relevance to cell cycle regulation and apoptosis. To harmonize some events of mitosis, ncRNA-guided tRNase Z^L^, the activity of which may be modulated by γ-tubulin and/or myosin II, may play a pivotal role through controlling the levels of mRNAs that produce key proteins for mitosis.

## Materials and Methods

### tRNase Z^L^ preparation

The histidine-tagged human full-length and Δ30 tRNase Z^L^ proteins were over-expressed from the expression plasmid pQE-80L (Qiagen) in *E. coli* strain Rosetta(DE3)pLysS (Novagen) and purified with nickel-agarose beads [6]. The human full-length and Δ30 tRNase Z^L^ proteins were also over-expressed from the expression plasmid pTYB11 (New England BioLabs) in the same strain and purified with chitin beads [5]. To check their purity, the proteins were separated on an SDS-7.5% polyacrylamide gel, and visualized by staining the gel with Coomassie brilliant blue R-250.

### RNA synthesis

Two target RNAs were synthesized with T7 RNA polymerase (Promega) from the corresponding synthetic DNA templates. The sequences of these RNAs are as follows: target-2, 5′-GAGCUAUUGCGCCGCUGGGUUGGAUUCCCAGCCAGGGAAGGCUUCU-3′; target-3, 5′-GAGGACUUUCUUGGUUUACUAUUG-3′. The transcription reactions were carried out under the conditions recommended by the manufacturer (Promega), and the transcribed RNAs were purified by denaturing gel electrophoresis.

The RNA transcripts for the target RNAs were subsequently labeled with fluorescein according to the manufacturer's protocol (GE Healthcare). Briefly, after the removal of the 5′-phosphates of the transcribed RNAs with bacterial alkaline phosphatase (Takara Shuzo), the RNAs were phosphorylated with T4 polynucleotide kinase (Takara Shuzo) and ATPγS. Then a single fluorescein moiety was appended onto the 5′-phosphorothioate site. The resulting fluorescein-labeled RNAs were gel-purified before assays.

The 5′-half-tRNA^Glu^ (5′-UCCCUGGUGGUCUAGUGGUUAGGAUUCGGCGCUCU-3′), the 28S rRNA fragment (5′-UUGAAAGUCAGCCCUCGACACAAGGGUUUG-3′), and the four siRNAs [34] targeting the human Ago2 mRNA were chemically synthesized by Nippon Bioservice. The siRNA targeting the human tRNase Z^L^ mRNA was obtained from Qiagen: sense, r(GACUCCGAGUCGAAUGAAA)d(TT); antisense, r(UUUCAUUCGACUCGGAGUC)d(TG).

### *In vitro* RNA cleavage assay

*In vitro* RNA cleavage assays [5], [6] for the fluorescein-labeled target RNAs (2 pmol) were carried out at 50°C in the presence of the unlabeled small ncRNAs (20 pmol) using histidine-tagged human tRNase Z^L^ (50 ng) in a mixture (6 µl) containing 10 mM Tris-HCl (pH 7.5), 1.5 mM dithiothreitol, and 3.3 mM MgCl_2_. After resolution of the reaction products on a 10–20% polyacrylamide-8 M urea gel, the gel was analyzed with a Typhoon 9210 (GE Healthcare).

### Cell culture

The various human cells were cultured in Dulbecco's modified Eagle's medium (DMEM; Sigma) supplemented with 10% fetal bovine serum (FBS; MP Biomedicals) and 1% penicillin-streptomycin (Invitrogen) at 37°C in 5% CO_2_ humidified incubator.

### Transfection

The 293 cells were transfected with plasmids or with plasmids and RNA using Lipofectamine 2000 (Invitrogen) according to the manufacturer's protocol. The cells were usually cultured for further 18 h, and the cells transfected with the tRNase Z^L^ siRNA or the Ago2 siRNAs were cultured for further 42 h.

### Fluorescent microscopic analysis

Human cells were cultured on a 12-mm-diameter coverslip coated with poly-D-lysine. For indirect fluorescent analysis, the cells were fixed with 4% paraformaldehyde in phosphate-buffered saline (PBS) for 10 min, permealized with HBS-PBS (5% horse serum, 1% bovine serum albumin and 0.1% saponin in PBS) for 15 min, and incubated with primary tRNase Z^L^ antibodies for 3 h, and with an Alexa488-conjugated secondary antibody (Invitrogen) for further 1 h. MitoTracker Red (Cambrex) was used to stain the mitochondria according to the manufacturer's protocol, and DAPI (Sigma) was to stain DNA. The cells were analyzed by the deconvolution method with the fluorescent microscope system Axio Imager.M2 (Zeiss).

### Western analysis

Whole cell extracts dissolved in a buffer (50 mM Tris-HCl pH 6.8, 2% SDS, 10% glycerol, 100 mM dithiothreitol) or the subcellular fractions described below were separated by SDS/7.5–15% polyacrylamide gel electrophoresis, and transferred to a nitrocellulose membrane. The membrane was probed with antibodies raised to a human tRNase Z^L^ peptide (amino acid 812–826), recombinant human tRNase Z^S^, or a human Ago2 peptide (amino acid 7–48; Upstate), or antibodies against the standard proteins calpain, porin, c-jun, and vimentin using the ECL Western Blotting Detection System (GE Healthcare).

### Subcellular fractionation

Four subcellular fractions, cytosolic, membrane/organelle, nuclear and cytoskeletal fractions, were prepared from 293 cells by using a ProteoExtract Subcellular Proteome Extraction Kit (Merckbiosciences).

### Luciferase assay

The modified luciferase expression plasmids pGL3-T(tRNA) and pGL3-T(rRNA), which produce mlucT(tRNA) and mlucT(rRNA), respectively, were constructed by inserting annealed synthetic double-stranded DNAs containing the sequences T(tRNA) (5′-CGCCGCTGGGTTGGATTCCCAGCCAGGGA-3′) and T(rRNA) (5′-GACTTTCTT-3′), respectively, between *Xba* I and *Fse* I sites of pGL3-Control vector (Promega).

The 293 cells were plated at densities of 2×10^5^ cells/ml on 24-well dishes in 500 µl/well DMEM supplemented with 10% FBS. After incubation at 37°C for 24 h, the cells were co-transfected with 0.2 µg/ml of one of the pGL3 series plasmids and 0.2 µg/ml of the β-galactosidase expression plasmid pTK-β (Clontech) together with chemically synthesized 5′-half-tRNA^Glu^ (100 nM), the 28S rRNA fragment (100 nM), the human tRNase Z^L^ or tRNase Z^S^ expression plasmid (0.5 µg/ml), the human tRNase Z^L^ siRNA (100 nM), or the four human Ago2 siRNAs (25 nM each). After further 18- or 42-h incubation, the cells were harvested, and the luciferase and β-galactosidase activities were measured using a PicaGene Kit (Toyo Ink) and a β-Gal Reporter Gene Assay Kit (Roche), respectively, with a Veritas Microplate Luminometer (Promega). The luciferase activity was normalized against the β-galactosidase activity.

### Real-time PCR for luciferase mRNA quantitation

The total RNAs from the 293 cells that were co-transfected with one of the pGL3 series plasmids (0.2 µg/ml) and the human tRNase Z^L^ expression plasmid (0.5 µg/ml) or the human tRNase Z^L^ siRNA (100 nM) were extracted with ISOGEN. The levels of luciferase and glyceraldehyde 3-phosphate dehydrogenase (GAPDH) mRNAs were quantitated by real-time PCR using a LightCycler 480 SYBR Green I Kit (Roche). The luciferase mRNA level was normalized against the GAPDH mRNA level. The primer pair for the luciferase mRNA was 5′-TCTGGATCTACTGGTCTGCCTAA-3′ and 5′-GCGCACTTTGAATCTTGTAATCCTG-3′, and that for the GAPDH mRNA was 5′-CTCTTGTGCTCTTGCTGGG-3′ and 5′-ACCCATCCTCCACCTTTG-3′.

### 3′ RACE

The total RNAs from the 293 cells that were transfected with one of the pGL3 series plasmids (0.2 µg/ml) were extracted with ISOGEN. The luciferase mRNA fragments generated by cleavages in its 3′ UTR were amplified by reverse-transcription PCR. After adding the linker 5′-r(UUU)d(AACCGCGAATTCCAG)-3′ to the 3′ ends, the fragments with the linker were subjected to reverse transcription, and the resulting cDNAs were amplified first using the primer pair 5′-ACGTCGCCAGTCAAGTAACA-3′ and 5′-GACTAGCTGGAATTCGCGGTTAAA-3′ and then using the primer pair 5′-TCCCCGCGGGGAAGGCCAAGAAGGGCGGAAAGA-3′ and 5′-GACTAGCTGGAATTCGCGGTTAAA-3′. The PCR products of ∼100 bp were recovered from an agarose gel, cloned into the TA cloning vector pGEM-T (Promega), and sequenced.

Likewise, 3′ RACE for the human PPM1F mRNA fragments was carried out. The cDNAs were amplified first using the primer pair 5′-CCCACAGAAGAGCAGCCCAA-3′ and 5′-GACTAGCTGGAATTCGCGGTTAAA-3′ and then using the primer pair 5′-TCCTGGACACGCTCCTGCAA-3′ and 5′-GACTAGCTGGAATTCGCGGTTAAA-3′.

### Co-immunoprecipitation

The 293 cell total extracts prepared basically as described previously [1] were incubated for 1 h with the human tRNase Z^L^ antibodies attached to Protein A sepharose (Sigma). As a negative control, bovine serum albumin was used instead of the antibodies. After washing the sepharose with a buffer (150 mM NaCl, 50 mM Tris-HCl, pH 7.4, 0.05% Nonidet P-40) 6 times, RNA components were extracted with phenol/chloroform, and precipitated with ethanol. The precipitated RNA samples were amplified by reverse-transcription PCR basically according to Pfeffer *et al*'s method [35], and cDNAs of ∼30 bp were cloned and sequenced.

### Northern analysis

RNAs from nuclei and cytoplasm of the 293 cells that were separated by using a Paris Kit (Ambion), and total RNA were extracted with ISOGEN. The RNA samples (4 µg of nuclear RNA, 24 µg of cytoplasmic RNA, and 30 µg of total RNA) were separated by 10% polyacrylamide/8 M urea gel electrophoresis, and electrically transferred to a Hybond N^+^ membrane (GE Healthcare). The DynaMarker small RNA II (BioDynamics) was used as RNA size standards after 5′-fluorescein-labeling. The membrane was ultraviolet-crosslinked, probed with a 5′-^32^P-labeled deoxyoligonucleotide in a QuickHyb buffer (Stratagene) at 45°C, and analyzed with a Typhoon 9210.

### DNA microarray analysis

Total RNA was extracted with ISOGEN from the 293 cells transfected with or without the human tRNase Z^L^ expression plasmid (0.5 µg/ml). The samples were hybridized to a GeneChip Human Genome U133 Plus 2.0 Array (Affymetrix), and analyzed using Microarray Suite version 5.0 according to the manufacturer's protocol.

### PCR analyses for endogenous mRNAs

Total RNA was extracted with ISOGEN from the 293 cells transfected with the 28S rRNA fragment (0.1 µM) or 5′-half-tRNA^Glu^ (0.1 µM) and/or the tRNase Z^L^ expression plasmid (0.2 µg/ml). For non-real-time PCR analysis, cDNA synthesis for 1 µg of the total RNA was carried out using a PrimeScript RT-PCR Kit (Takara Shuzo), and PCR for the cDNA was using Ex Taq DNA polymerase (Takara Shuzo) and each mRNA (or rRNA) specific primer pair (Table S3). PCR products were separated on a 1.5% agarose gel and stained with ethidium bromide.

For real-time PCR analysis, mRNA and 28S rRNA levels were quantitated using a Transcriptor First Strand cDNA Synthesis Kit (Roche), a LightCycler Taqman Master (Roche), and each mRNA (or rRNA) specific primer pair and probe (Table S3). The first strand cDNA synthesis for mRNA was performed using an oligo(dT)_18_ primer. The mRNA level was normalized against the 28S rRNA level.

### Statistical analysis

Differences between control and experimental groups were evaluated by the Student's *t*-test.

### Accession numbers

Small ncRNA sequences were deposited in the DNA Data Bank of Japan (www.ddbj.nig.ac.jp), accessions AB330769–AB330794, and microarray data were in Gene Expression Omnibus (http://www.ncbi.nlm.nih.gov/geo), accession GSE12524.

## Supporting Information

Figure S1 Northern blotting for small ncRNAs co-immunoprecipitated with human tRNase Z^L^. The probe sequence of each ncRNA is indicated in Table S1. Vertical bars denote the small ncRNAs. The nuclear and cytoplasmic RNAs were probed for U3 snRNA to evaluate the integrity of the nuclear/cytoplasmic fractionation. U3 snRNA probe sequence, 5′-GACCGCGTTCTCTCCTTCTCACTCCCCAAT-3′; T, total RNA; N, nuclear RNA; C, cytoplasmic RNA. The presence of a smaller amount of 5′-half-tRNA^Glu^ in the total RNA of 293 cells than in the fraction RNAs is because the total RNA was prepared from the cells with a lower density (Figure S8A).
(0.08 MB PDF)

Figure S2 Differential interference contrast (DIC) and fluorescent (Alexa488) microscopic images of human 293 kidney cells, A549 epithelial lung cells, HepG2 hepatoma cells, and IMR90 lung fibroblasts. The pictures in the right panels, which are negative controls for the ones in Figure 3, were taken under the conditions that the cells were incubated with an Alexa488-conjugated secondary antibody without being incubated with primary tRNase Z^L^ antibodies. Bar, 20 µm.
(1.48 MB PDF)

Figure S3 DNA microarray analysis. (A) mRNAs downregulated by tRNase Z^L^ overexpression. (B) and (C) The KEGG pathway analysis. The p53 signaling pathway (B) and apoptosis (C) are shown. Red and blue squares denote genes upregulated and downregulated, respectively, by tRNase Z^L^ overexpression.
(0.38 MB PDF)

Figure S4 Reverse-transcription PCR analyses for selected mRNAs. Total RNA samples were prepared from the 293 cells that were transfected with the 28S rRNA fragment (+rRNA), 5′-half-tRNA^Glu^ (+tRNA), or the tRNase Z^L^ expression plasmid (+Z^L^). The numbers of potential target sites in the mRNAs for the 28S rRNA fragment and 5′-half-tRNA^Glu^ are shown.
(1.88 MB PDF)

Figure S5 Possible secondary structures of 5′-half-tRNA^Glu^/PPM1F mRNA complexes. Arrows denote expected cleavage sites. Numbers on the PPM1F mRNA are from the numbering system of the PPM1F mRNA sequence (GenBank accession NM_014634).
(0.22 MB PDF)

Figure S6 Distribution of 3′ ends of 5′ cleavage products of the human PPM1F mRNA. The 3′ ends were determined by 3′ RACE for total RNA from the 293 cells. Red line, expected cleavage site. Numbers on the PPM1F mRNA are from the numbering system of the PPM1F mRNA sequence (GenBank accession NM_014634).
(0.21 MB PDF)

Figure S7 Conservation of potential ncRNA-guided tRNase Z^L^ target sequences among human, mouse, and rat. (A) Comparison of potential 5′-half-tRNA^Glu^-guided tRNase Z^L^ target sequences in the human PPM1F mRNA with the corresponding sequences in the mouse and rat PPM1F mRNAs. Mouse and rat PPM1F mRNA sequences corresponding to the third target sequence in the 3′ UTR of the human PPM1F mRNA are not discernible. (B) Comparison of potential 28S-rRNA-fragment-guided tRNase Z^L^ target sequences in the human DYNC1H1 mRNA with the corresponding sequences in the mouse and rat DYNC1H1 mRNAs. Potential ncRNA-binding nucleotides and potential nucleotides that form T-stem-like structures are shown in red and in blue, respectively. Nucleotides in the mouse and rat sequences that are different from those in the human sequences are underscored. Numbers on the human PPM1F and DYNC1H1 mRNA sequences are from the numbering systems of the PPM1F mRNA sequence (GenBank accession NM_014634) and the DYNC1H1 mRNA sequence (GenBank accession NM_001376), respectively.
(0.21 MB PDF)

Figure S8 Northern analyses for small ncRNAs that work as sgRNAs. (A) Time course analysis for the levels of the 5′-half-tRNA^Glu^ (vertical bar) in 293 and HeLa cells, which were plated at the densities of 2×10^5^ and 1×10^5^ cells/ml, respectively. (B) Time course of the level of the RNase 65 RNA component 3′-truncated tRNA^Arg^ in human BJAB cells cultured in RPMI medium containing 10% FBS after adding 5-fluorouracil (400 µM). The tRNA^Arg^ probe sequence, 5′-GAATCTTCTGATCCGTAG-3′.
(0.49 MB PDF)

Table S1 Small RNAs co-immunoprecipitated with human tRNase Z^L^.
(0.02 MB PDF)

Table S2 Potential mRNA targets of tRNase Z^L^ guided by 5′-half-tRNA^Glu^.
(0.17 MB PDF)

Table S3 PCR primers and probes for endogenous mRNA analyses.
(0.05 MB PDF)
