# Supplementary figures and images for: Modulation of Gene Expression by Human Cytosolic tRNase ZL through 5′-Half-tRNA
Source: PLoS One. 2009 Jun 15;4(6):e5908. doi: 10.1371/journal.pone.0005908 (PMC2691602; doi:10.1371/journal.pone.0005908)

Figure S1

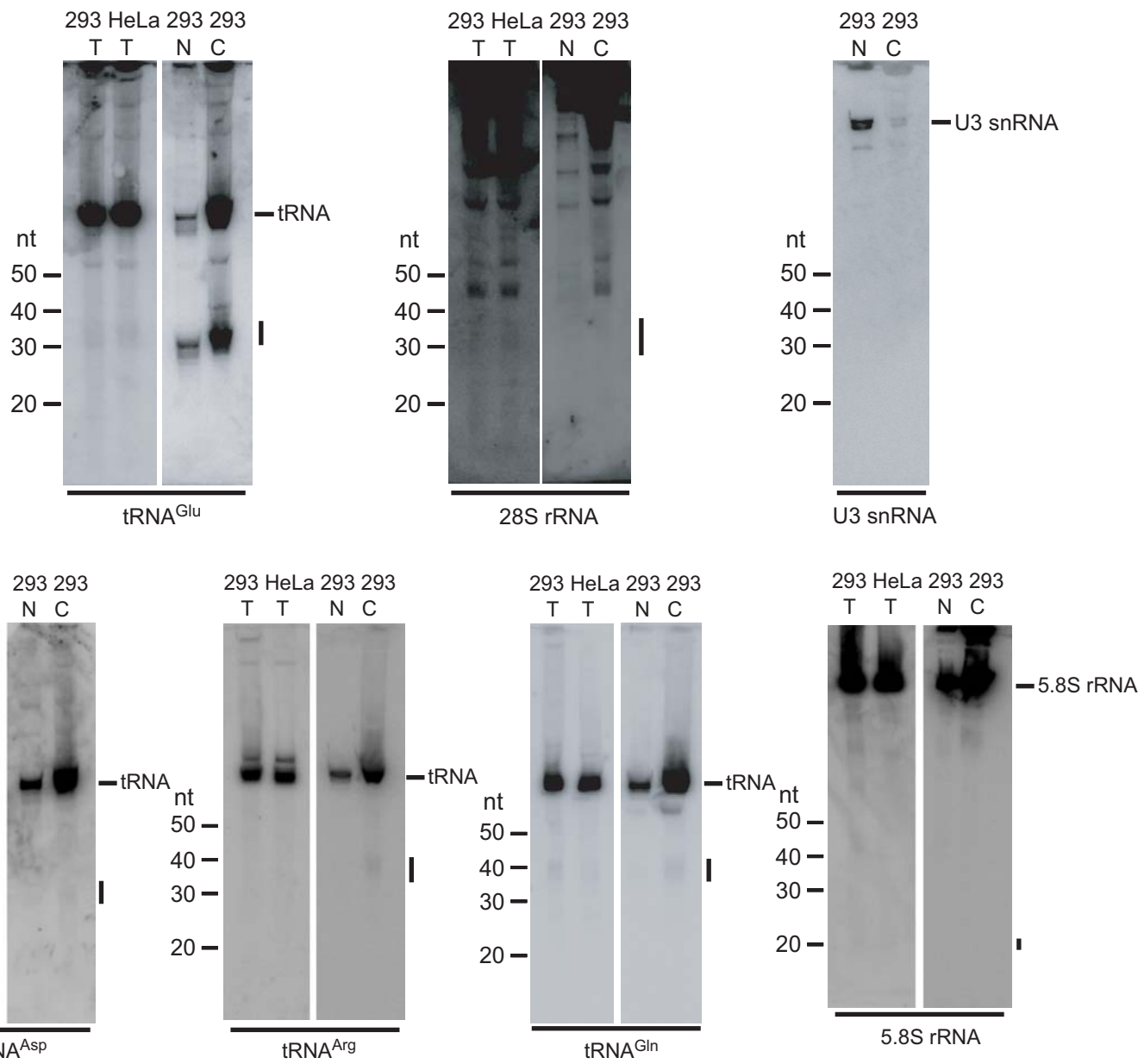

Supplement: Figure S1 — Northern blotting for small ncRNAs co-immunoprecipitated with human tRNase ZL. The probe sequence of each ncRNA is indicated in Table S1. Vertical bars denote the small ncRNAs. The nuclear and cytoplasmic RNAs were probed for U3 snRNA to evaluate the integrity of the nuclear/cytoplasmic fractionation. U3 snRNA probe sequence, 5′-GACCGCGTTCTCTCCTTCTCACTCCCCAAT-3′; T, total RNA; N, nuclear RNA; C, cytoplasmic RNA. The presence of a smaller amount of 5′-half-tRNAGlu in the total RNA of 293 cells than in the fraction RNAs is because the total RNA was prepared from the cells with a lower density (Figure S8A). (0.08 MB PDF) [file pone.0005908.s001.pdf]

Figure S2

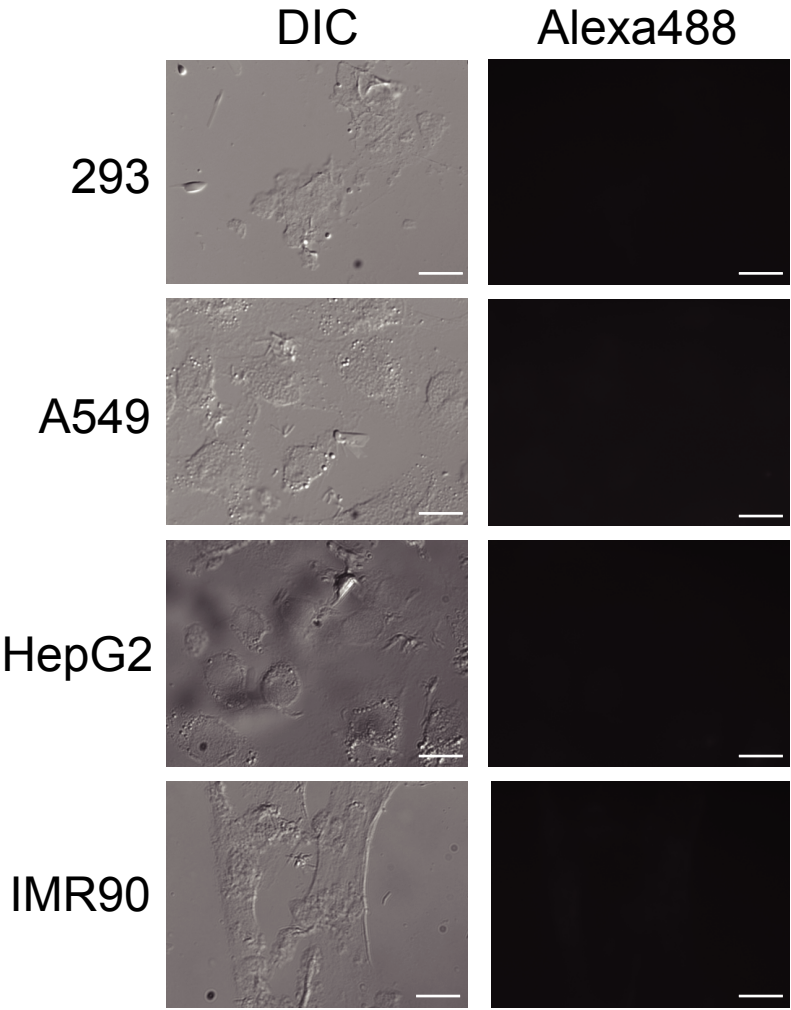

Supplement: Figure S2 — Differential interference contrast (DIC) and fluorescent (Alexa488) microscopic images of human 293 kidney cells, A549 epithelial lung cells, HepG2 hepatoma cells, and IMR90 lung fibroblasts. The pictures in the right panels, which are negative controls for the ones in Figure 3, were taken under the conditions that the cells were incubated with an Alexa488-conjugated secondary antibody without being incubated with primary tRNase ZL antibodies. Bar, 20 µm. (1.48 MB PDF) [file pone.0005908.s002.pdf]

Figure S4

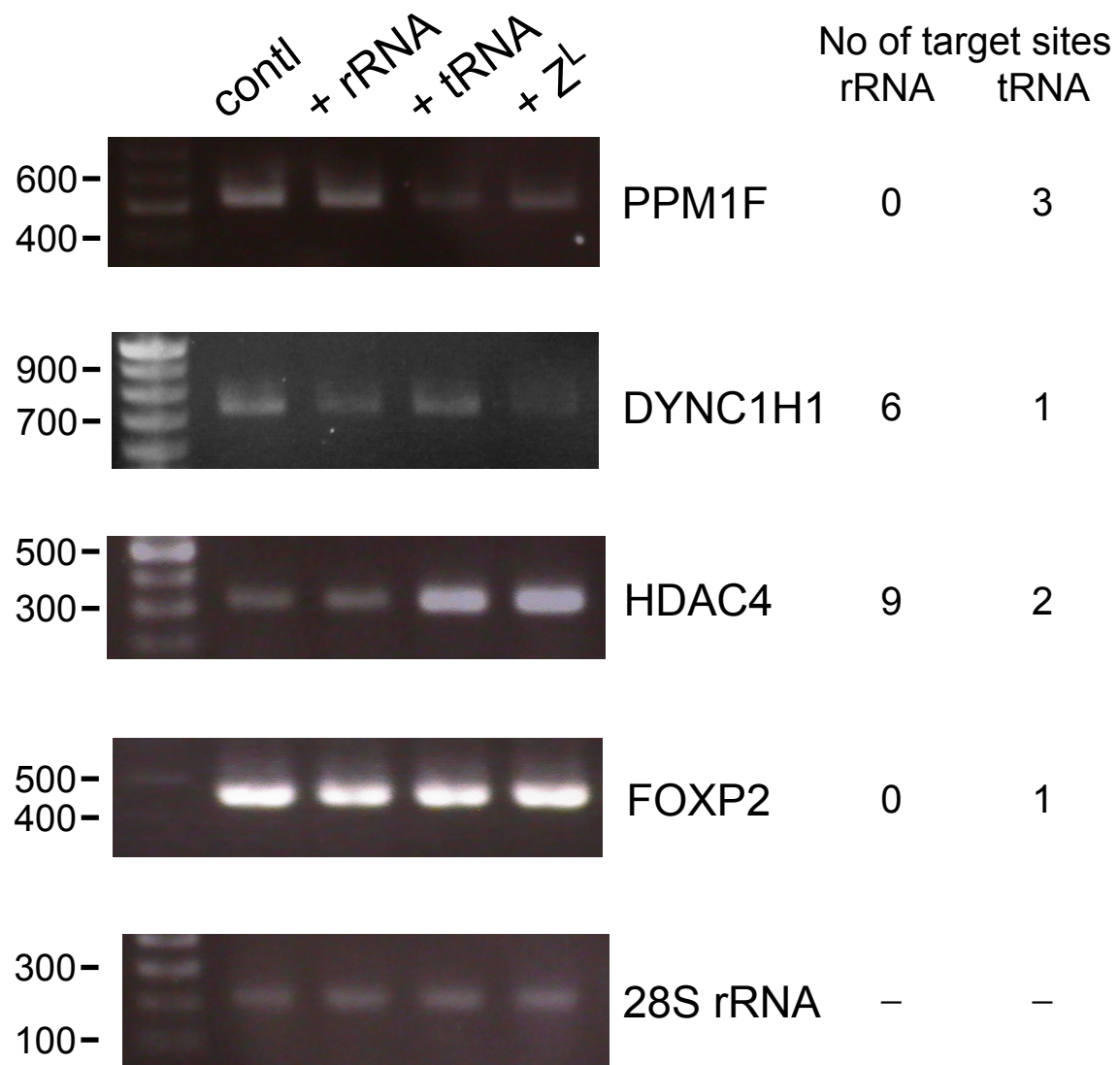

Supplement: Figure S4 — Reverse-transcription PCR analyses for selected mRNAs. Total RNA samples were prepared from the 293 cells that were transfected with the 28S rRNA fragment (+rRNA), 5′-half-tRNAGlu (+tRNA), or the tRNase ZL expression plasmid (+ZL). The numbers of potential target sites in the mRNAs for the 28S rRNA fragment and 5′-half-tRNAGlu are shown. (1.88 MB PDF) [file pone.0005908.s004.pdf]

Figure S5

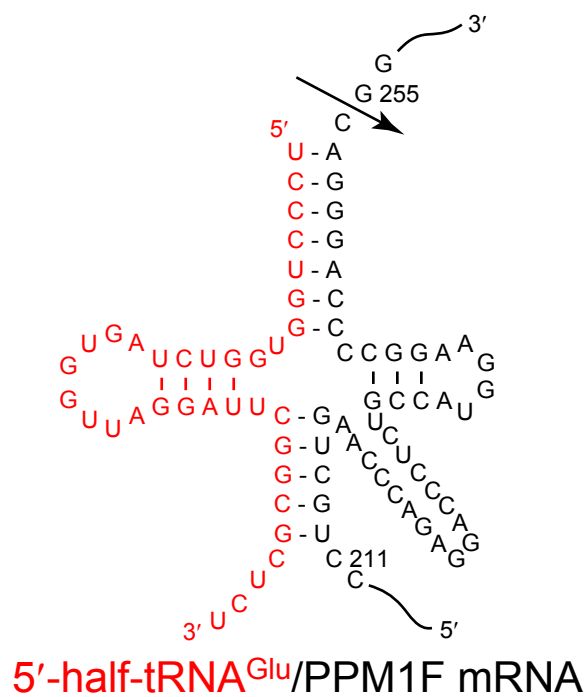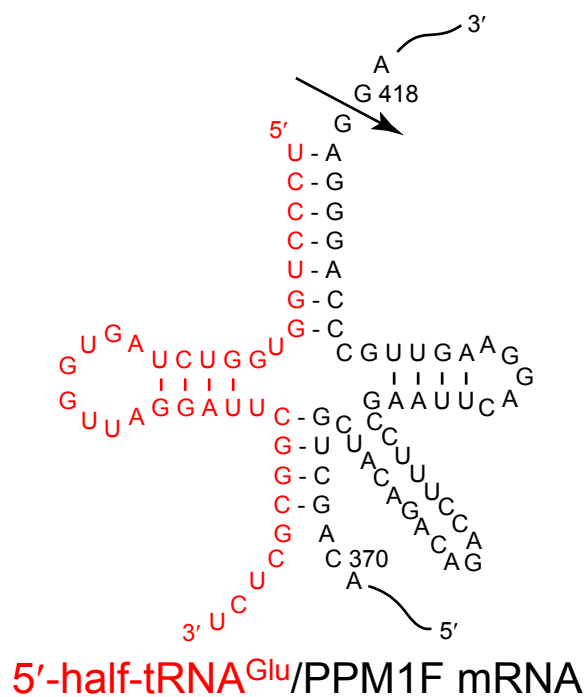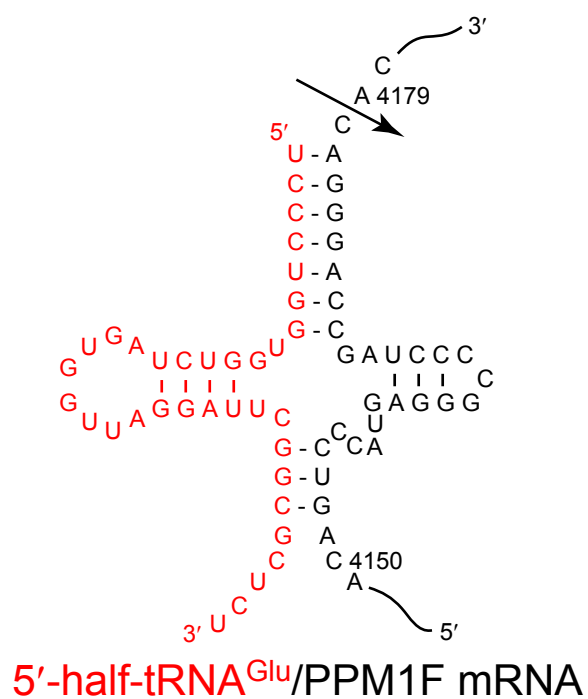

Supplement: Figure S5 — Possible secondary structures of 5′-half-tRNAGlu/PPM1F mRNA complexes. Arrows denote expected cleavage sites. Numbers on the PPM1F mRNA are from the numbering system of the PPM1F mRNA sequence (GenBank accession NM_014634). (0.22 MB PDF) [file pone.0005908.s005.pdf]

Figure S6

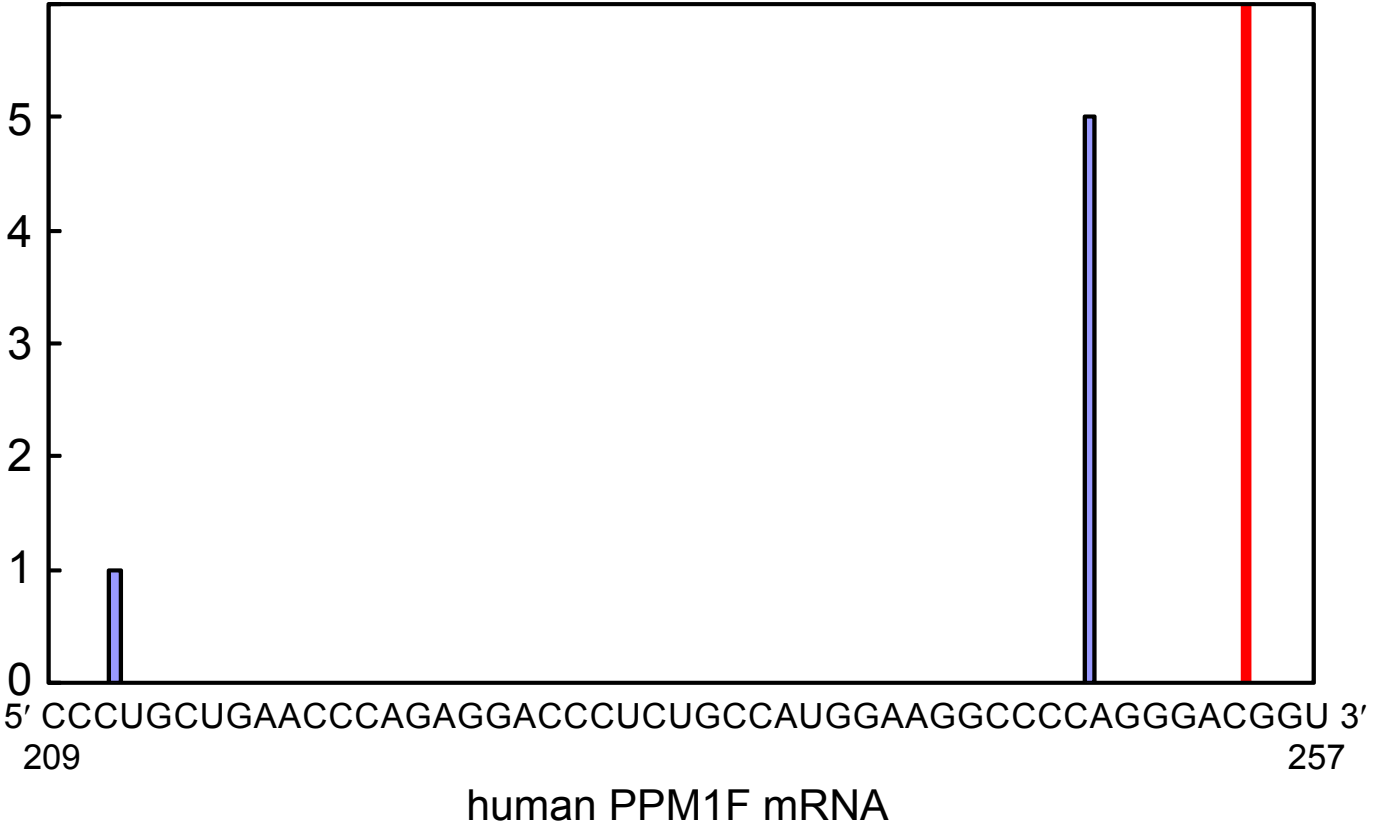

Supplement: Figure S6 — Distribution of 3′ ends of 5′ cleavage products of the human PPM1F mRNA. The 3′ ends were determined by 3′ RACE for total RNA from the 293 cells. Red line, expected cleavage site. Numbers on the PPM1F mRNA are from the numbering system of the PPM1F mRNA sequence (GenBank accession NM_014634). (0.21 MB PDF) [file pone.0005908.s006.pdf]

Figure S8

A

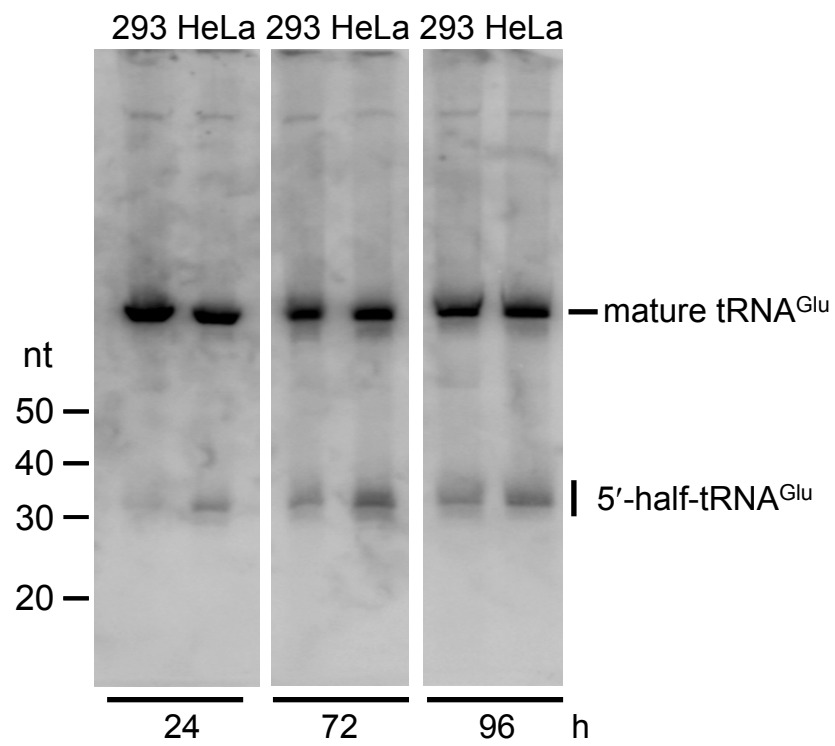

B

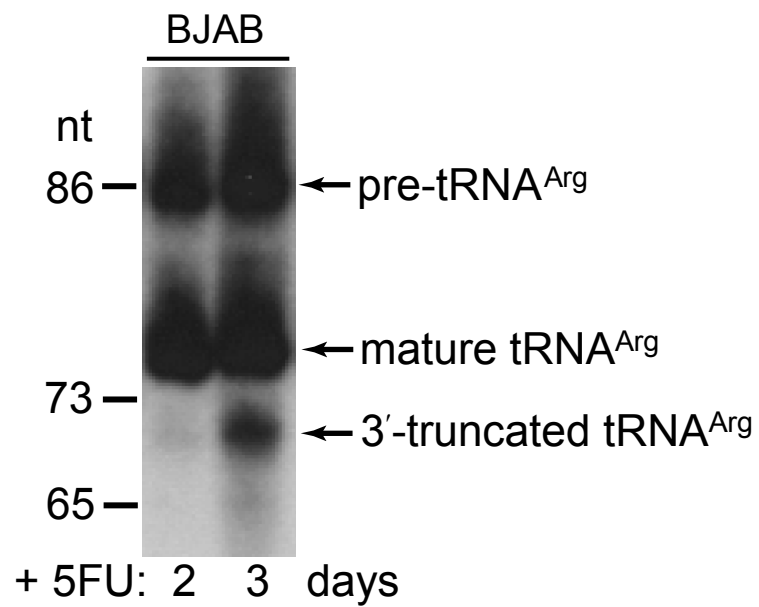

Supplement: Figure S8 — Northern analyses for small ncRNAs that work as sgRNAs. (A) Time course analysis for the levels of the 5′-half-tRNAGlu (vertical bar) in 293 and HeLa cells, which were plated at the densities of 2×10^5^ and 1×10^5^ cells/ml, respectively. (B) Time course of the level of the RNase 65 RNA component 3′-truncated tRNAArg in human BJAB cells cultured in RPMI medium containing 10% FBS after adding 5-fluorouracil (400 µM). The tRNAArg probe sequence, 5′-GAATCTTCTGATCCGTAG-3′. (0.49 MB PDF) [file pone.0005908.s008.pdf]
