# Supplementary material for: Modulation of Gene Expression by Human Cytosolic tRNase ZL through 5′-Half-tRNA
Source: PLoS One. 2009 Jun 15;4(6):e5908. doi: 10.1371/journal.pone.0005908 (PMC2691602; doi:10.1371/journal.pone.0005908)

Figure S3

A

List of mRNAs Downregulated by tRNase Z^L Overexpression

APP B3GNT5 CDC42SE2 DNAJC7 DYNC1H1 EIF4B GATA4 GNAS GTF3C2 HIST1H2AI HSPA4 KIF1A LETM1 LOC647190 LYPLA2 MED16 MT1F MXD4 PACS2 PBX2 PCSK9 PPM1F RBM6 RBM27 SAMD5 SEC14L1 SFN SFT2D2 SMCHD1 SREBF2 SRRM2 SYNE2 SYT6 TMED4 TMPO TNPO2 TRIM59 ZDHHC20 ZFAND6 ZGPAT ZNF609

B

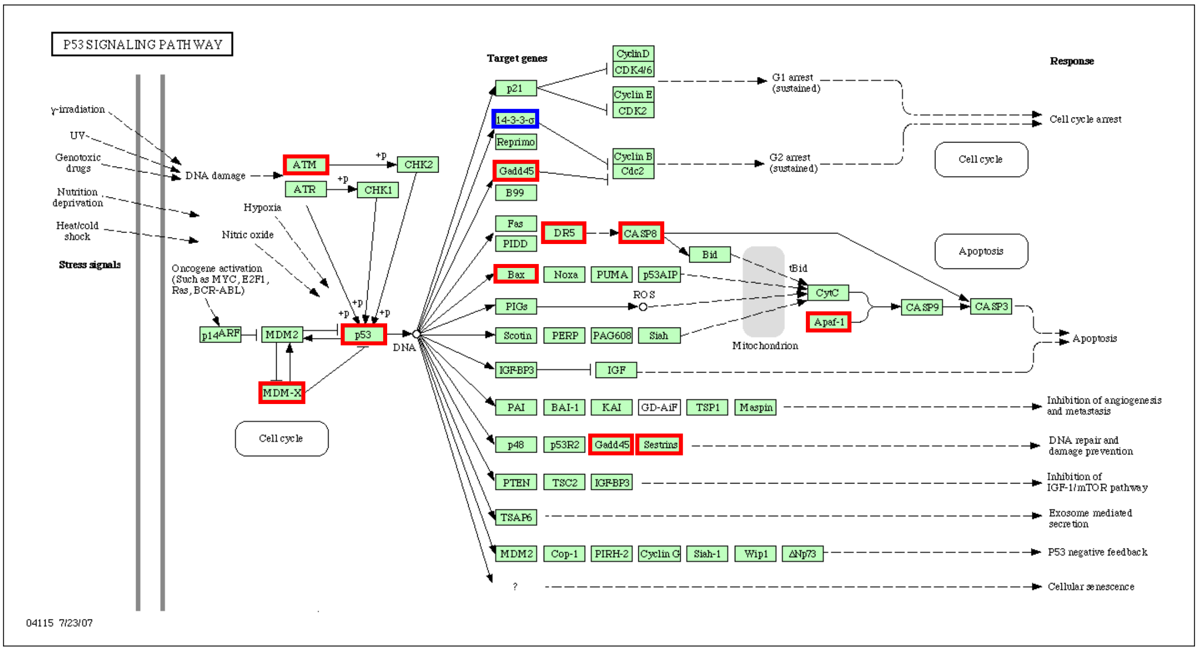

C

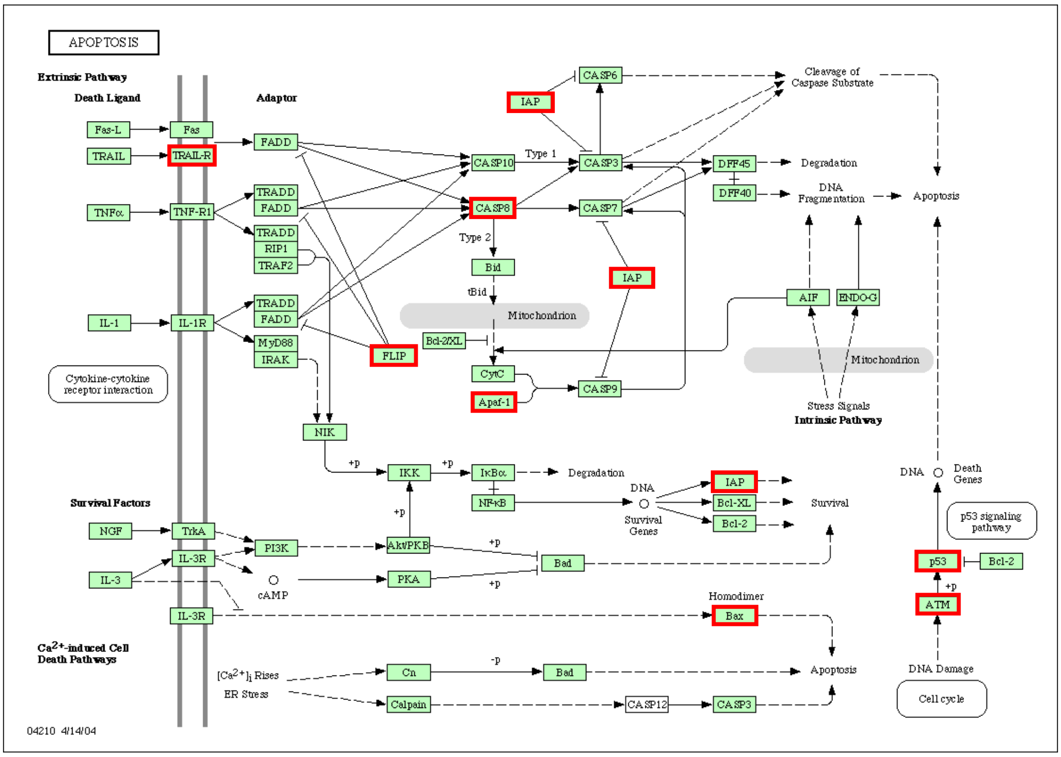

Supplement: Figure S3 — DNA microarray analysis. (A) mRNAs downregulated by tRNase ZL overexpression. (B) and (C) The KEGG pathway analysis. The p53 signaling pathway (B) and apoptosis (C) are shown. Red and blue squares denote genes upregulated and downregulated, respectively, by tRNase ZL overexpression. (0.38 MB PDF) [file pone.0005908.s003.pdf]
