# Supplementary material for: Modulation of Gene Expression by Human Cytosolic tRNase ZL through 5′-Half-tRNA
Source: PLoS One. 2009 Jun 15;4(6):e5908. doi: 10.1371/journal.pone.0005908 (PMC2691602; doi:10.1371/journal.pone.0005908)
Supplement: Figure S7 — Conservation of potential ncRNA-guided tRNase ZL target sequences among human, mouse, and rat. (A) Comparison of potential 5′-half-tRNAGlu-guided tRNase ZL target sequences in the human PPM1F mRNA with the corresponding sequences in the mouse and rat PPM1F mRNAs. Mouse and rat PPM1F mRNA sequences corresponding to the third target sequence in the 3′ UTR of the human PPM1F mRNA are not discernible. (B) Comparison of potential 28S-rRNA-fragment-guided tRNase ZL target sequences in the human DYNC1H1 mRNA with the corresponding sequences in the mouse and rat DYNC1H1 mRNAs. Potential ncRNA-binding nucleotides and potential nucleotides that form T-stem-like structures are shown in red and in blue, respectively. Nucleotides in the mouse and rat sequences that are different from those in the human sequences are underscored. Numbers on the human PPM1F and DYNC1H1 mRNA sequences are from the numbering systems of the PPM1F mRNA sequence (GenBank accession NM_014634) and the DYNC1H1 mRNA sequence (GenBank accession NM_001376), respectively. (0.21 MB PDF) [file pone.0005908.s007.pdf]

Figure S7

A

PPM1F mRNA

```
      210                                        255
human CCTGCTGAACCCAGAGGACCCTCTGCCATGGAAGGCCCCAGGGACG
mouse CCCACTGAGCCTGGAGAGCCCTTTGCCATGGAAGGTCCCAGGGACA
rat   CCCACTGAGCCTGGAGAGCCCTTTGCCATGGAAGGTCCCAGGGACA

      370                                           418
human CAGCTGCTACAGACAGACCTTTCCGAATTCAGGAAGTTGCCCAGGGAG
mouse CAGCTGCTGCAGACAGACCTTTCTGAATTTAAGAGGTTGCCTGAACAA
rat   CAGCTGCTTCAGACAGACCTCTCTGAATTTAAGAGGTTGCCTGAACAA
```

B

DYNC1H1 mRNA

```
         853      862   2375     2384   4167     4176
human    CAGACTTTGG        TAACTTTCTT        GAACTTTCTA
mouse    CAGACTTTGG        GAACTTCCTT        GAGCTTTCTA
rat      CAGATTTCGG        GAACTTCCTT        GAGCTTTCTA

        8061     8070   9073     9082  10645    10654
human    AAGACTTTTG        AAGACTTTGA        AAACTTTCAA
mouse    AAGACGTTTG        AAGACTTCGA        AAACTTTCAA
rat      AAGACGTTTG        AAGACTTTGA        AAACGTTCAA
```
