# Supplementary material for: Modulation of Gene Expression by Human Cytosolic tRNase ZL through 5′-Half-tRNA
Source: PLoS One. 2009 Jun 15;4(6):e5908. doi: 10.1371/journal.pone.0005908 (PMC2691602; doi:10.1371/journal.pone.0005908)
Supplement: Table S1 — Small RNAs co-immunoprecipitated with human tRNase ZL. (0.02 MB PDF) [file pone.0005908.s009.pdf]

Table S1. Small RNAs Co-immunoprecipitated with Human tRNase Z^L

annotation 1		annotation 2	sequence (5′ to 3′)
tRNA	5′-half-tRNA^{Glu}	piRNA R-72193	TCCCATATTG TCTAGCGGTT AGGATTCCTG GT
	5′-half-tRNA^{Glu}	piRNA R-60565	TCCCTGGTGG TCTAGTGGTT AGGATTCGGC GCTCT
	3′-half-tRNA^{Arg}	piRNA R-30229	CTGGGGATTG TGGGTTCGTG TCCCATCTGG GTC
	3′-half-tRNA^{Arg}	piRNA R-30229	GCTGGGGATT GTGGGTTCGT GTCCCATCTG GGTCGC
	3′-half-tRNA^{Asp}		AAGGTTGCCT GTTCATATCA CGTCGGGGTC AC
	3′-half-tRNA^{Asp}		TGTCACGCGG GAGACCGGGG TTCGATTCCC CGACGGGGA
	3′-half-tRNA^{Gln}		GAATCCAGCG ATCCGAGTTC GTGTCTCGGT GGAACCT
	3′-half-tRNA^{Gln}		TGAATCCAGC GATCCGAGTT CATGTCTCGG TGGAACCT
	3′-half-tRNA^{Gly}	piRNA R-36173	CACGCGGGAG GCCCGGGTTC GTTTCCCGGC CAATGCACC
	3′-half-tRNA^{Met}	piRNA R-33536	TCATAATCTG AAGGTCGTGA GTTCGCTCCT CACACGGGGC AC
	3′-half-tRNA^{Trp}		TGGGAGACCG GGGTTCGATT CCCCGACGGG G
rRNA	5.8S rRNA fr.	piRNA R-63876	TGACTCTTAG CGGTGGATCA CT
	5.8S rRNA fr.	piRNA R-63876	TCGTACGACT CTTAGCGGTG GAT
	18S rRNA fr.	piRNA R-84460	TCTAAGTACG CACGGCCGGT ACAG
	28S rRNA fr.	piRNA R-16	AGTCTGCCCT CGACACAAGG GTTTGT
	28S rRNA fr.	piRNA R-16	TTGAAAGTCA GCCCTCGACA CAAGGGTTTG
	28S rRNA fr.	piRNA R-63455	CGCGACCTCA GATCAGACGT GGCGACCCGC TGAA
snRNA	U2 snRNA fr.		ATTGCAGTAC CTCCAGGAAC GGTGCAC
	U3 snRNA fr.		ATTGGGGAGT GAGAAGGAGA GAACGCGGTC TGAA
	U5 snRNA fr.		TACTAAAGAT TTCCGTGGAG AGGAACAACT CTGAGT
	U6B snRNA fr.	β-actin-like protein	CGTGAAGCGT TCCATATTTT T
mRNA	DOCK9 intron fr.		CCAGCGCCGG TCCTGCTGTA TTTGGAAACG CAC
unknown			TTCAGATGGA CTTCCGTGCT T
			GGACCTCTCA CACACAGTTC GC
			TTGGAGTCCG TGGATATTTA ATTTTTCTGA TCC
			CGGAATACGA GCCCCATTCA GGTTGCTGGA TGC

Sequences of DNA probes used for northern blotting are complementary to underscored ones.
