# Supplementary material for: Modulation of Gene Expression by Human Cytosolic tRNase ZL through 5′-Half-tRNA
Source: PLoS One. 2009 Jun 15;4(6):e5908. doi: 10.1371/journal.pone.0005908 (PMC2691602; doi:10.1371/journal.pone.0005908)
Supplement: Table S2 — Potential mRNA targets of tRNase ZL guided by 5′-half-tRNAGlu. (0.17 MB PDF) [file pone.0005908.s010.pdf]

Table S2. Potential mRNA Targets of tRNase Z^L Guided by 5′-half-tRNA^{Glu}

ABCB8 (NM_007188)

621 tctcagcacccac ctg ct tatc ctctat ggtg t ccaggga ctgctgacct

2581 cagtctccc tgcc ccacccagcagcttcaaattggcca ggccta cag tagct ccaggga a

ABCF1 (NM_001090)

1851 g tgctg ggtctgca tggtgtg acatt cggcta ccaggga c

ABL1 (NM_007313)

2131 tgacgtgcctgaga tgcc tca ctc caa ggg ccaggga gag

3101 cc cgccg aggagtccagagtgaggaggcacaagc actc ctct gagt cg ccaggga gggac

ABR (NM_001092)

3841 gggttc tgctg gggcctccttgtt ggtgct gta ggcacc cg ccaggga gcagggacccga

ACP6 (NM_016361)

51 aatcgagtagtcct tgc tggtgg agccg ctgccg ccaggga actcagggc

211 gagcgaccaacc ccg accc gggt taaa actc ccaggga ct

ADCK2 (NM_052853)

2261 aaggg gcc tatccattc cattg tggaagctgggc caggtg ccaggga cactctccttcag

AGMAT (NM_024758)

961 tcagctttgata ttg a cgctctgg atcctg cctatgcg ccaggga caggg

AGRN (NM_198576)

2251 ccaggtgtga gtc acagcgagg gct ctacgtagc ggc ccaggga gcctgc

AKAP8L (NM_014371)

71 cgtcgtcggatgttg tgttg cccgccacc atgagct acaca ggctttgt ccaggga tctg

AKAP9 (NM_147166)

5671 ggaaatcggaagtagtat gct tct gaa actc ttt ccaggga aacatctct

ALAD (NM_001003945)

2341 agggtgagccatcaa gct aagac aat tggggaagagc att ccaggga gag

ALPK2 (NM_052947)

3181 aacaacattaac tgct aataatgag tgct ttca agcg a ccaggga gactgaggacacatc

5271 agagagagaagtcc ctg gaagcc cga gcaggcaaa tcg ccaggga ccctc

5741 tttgccatcgtgcaa gcc ag tcc gaa gga ccaggga ctct

6741 cctaatg gcc ac tgg ctagcagcacacaatc tcg ccaggga aaatctgag

AMT (NM_000481)

871 ctg aaaaacccagaggtgaag ctg gcagggctgg cag ccaggga cagcct

1211 cgatgggttatg tgcc ct gcga gtacag tcgt ccaggga c

ANAPC2 (NM_013366)

1511 tgggtcccggacc ctg tgg atg ccg at ccaggga agtcga

ANKRD13A (NM_033121)

431 cgagtcttact ccg acataaagca gat gtgacaaaagaaa atc g ccaggga

2141 aggcaacaac tgcc ccttcttta tgca gagg tgca gaa ccaggga ctcct

2191 gggcccatccag gctg c tccct ggggtggaga aggga ccaggga ttgcag

ANKRD52 (NM_173595)

7461 gcc ctttccctggggaatctg gaag cctga cttt gccc ccaggga agttg

ANTXR2 (NM_058172)

61 actcctagtttgttc tgc cgt cgcc gcg tc ccaggga ccccttgtcccga

APPL1 (NM_012096)

1531 tgtgaagatcagcctg gcc agg caaa agcc tttg g ccaggga ggcaggcg

4831 cca gtt attccttctacctttaaaaatt ttg agaacttgc caa ccaggga ttaaagctat

APOF (NM_001638)

481 gaagccctg gcc tctgct ctg cagctgt tag ccaggga gcagcaaagcac

651 gtattatgctactcaaaa ctg cct gggca agg ccaggga acgaggccgag

AP3D1 (NM_003938)

3691 gcctgcgagcc ccg caccacccc gcg gagca cgt ac ccaggga ccgcagc

ARHGAP6 (NM_013427)
2281 aaagagttcctgagggaca tgcc aga ccccct tct ca ccaggga gctgta
3501 tgacaagcg gcc ccc gcct ccataccc gggc ccaggga ag

ARHGEF18 (NM_015318)
3391 ctcaaggaaa gtt tttaatggaaag ttg agccagaac taa a ccaggga gc

ARSD (NM_009589)
81 gatc cgccg cgcggaggggac gcg ccgcgcc cgc cg ccaggga ctctttg

ASCL1 (NM_004316)
1 cttctggccagggaacgtggaagg cgc a ccg acagggatc cgg ccaggga

ASPM (NM_018136)
9251 cagaaatc tgctg c tttgatcata caaaaa tatatacgag ccaggga ggc

ATG4A (NM_052936)
31 tagcag tgc aga act aca agt c ccaggga t

ATHL1 (NM_025092)
541 cccagacctggacc tgc atca gggt cctg actt ccaggga gcccggtacc
1091 atccgca cgctg gacggggccctggagaac gcccag aac ctgggc ta ccaggga gcc
1321 tgtggccgagttttggtgca gtcg t gtt gagtgg agc c ccaggga ggaaa

ATP11A (NM_032189)
3911 ccatcattggcctt tgctg tcac tgg gagagaagag ccg t ccaggga ccc

AXIN2 (NM_004655)
2071 caaa cgc aatgggaaaggcacggagc cgggc ctggccct gcccg ccaggga

AXL (NM_001699)
271 caggggcacgcag gctg aagaaagt ccc ttcgt ggg caac ccaggga ata
2231 gacttcgg gct ctccaagaagatctacaa tgggg acta ctaccg ccaggga cgtatcgcc
2411 tatccggg cgt ggagaacagcg agat ttatgact atct gcg ccaggga aa

AZI1 (NM_014984)

3401 gc tgctg gagcagcacaggaggcc cac gccaagtaccaa gtg a ccaggga

BAT2 (NM_080686)
3011 gcacagagacc cgc tggg gcc ctcg t ccaggga gcagtcg

BCHE (NM_000055)
691 tgcc ctaggattctt agctttg ccaggaaatcc tgaggct ccaggga aca

BCL2L11 (NM_138621)
2281 cacactggtca gtc atttcagag gca gcaga tgc ccaggga gacccaaga

BCR (NM_021574)
4921 ctcagaaggcctgtgaaa tgtc ag ggg acagga ccc ccaggga gggaacc

BLOC1S3 (NM_212550)
581 ctggctgccgcctgc tgccg ga cat ccgcggc gtg ccaggga ccgagcct

BMPER (NM_133468)
311 cggca gctg agcagaggcgg cggcgcgg gac ctgcagtcg ccaggga ttc

BMP8B (NM_001720)
181 agccaggacagg tgtc gcgc ggcgg gcgc t ccaggga ccg

BNC1 (NM_001717)
3601 tcccctca gcc ctgcaccaga ggt ctcatatgtt acc ccaggga gttctc

B3GNT3 (NM_014256)
1331 caggaagctgagacctttgtggt ctg ag cat aaggga gtg ccaggga agg

B4GALNT3 (NM_173593)
1051 aaacccagctat ctg gtggatg ggctt cctctgc agcgct a ccaggga ct

B4GALNT4 (NM_178537)
61 ctgctgagctg cgccg cgtggctcaccta cgtgc acctgggcctg gtgcg ccaggga cgc

C1QL2 (NM_182528)

1821 ctagaagcggagg gct ccgg gcc ctgg ccaggga ggtagg

CACNA1C (NM_000719)
6311 ctcatgg tgcc cagcca ggc tggg gcc ccaggga ggcagt

CACNA1E (NM_000721)
7511 gcacaggctaa gtc aggaa cat cgg atg ccaggga gggag

CACNA1S (NM_000069)
181 agca ctg gggtgggggagcagga gaa agccaga ttc ccaggga agccatgg

CALN1 (NM_001017440)
3371 ataggggtgcaggaccctaagtaga ctg ggt agaa acc ccaggga tgatt
8431 gctgag tgctg agcggtgcctgcttttga tgtgg cccac ccatg a ccaggga gagagggc

CAND2 (NM_012298)
1081 ggtcaaggccctgcagcggca gct taaagat cggagc gtcaga gcccg ccaggga tgctt
3521 tgggggccc tgtcg ctcctggtc aggg cttacagtgcc ttct ccaggga c
3931 aatcagaaat ttg gaagatgatac ggg tttcttt ttt ccaggga ggagga

CA10 (NM_001082534)
1 acagttcccaatcgggacaagcg cgccg a gaaatc ctg gatcttc ccaggga ttccctct

CCNF (NM_001761)
301 tgtgtgggcatg tgcc agcttcc agg agctgtggccg tct ccaggga acc

CD177 (NM_020406)
521 cag gct caggggaggaggcatc ttctc caatct gagagt ccaggga tgca

CD2AP (NM_012120)
2361 tgtcc tgtc ttcttgagtggtgtg gacct ggtgttcata atgtt ccaggga ttcagaagc

CD44 (NM_000610)
361 tc cgccg gcccc tgc ccc gcg c ccaggga t

CDC25A (NM_201567)

2331 aaatac tgcc attctaggtag ggt tttatt atc ccaggga
2701 gggccacccccggctcagtgctggaa tgttg c agt gtag gtt tc ccaggga aggggggtg

CDC42BPG (NM_017525)
3031 gtcctggcct ctg at gtt atccatgccc aat ccaggga cc
4331 accacctagtacacgtgggccc tgcc aa cggg cgg cccg gcg ccaggga c

CECR2 (NM_031413)
1681 agacggagtcgg gctg ggcgaagtggtggg agc catgtttggaccc gct ccaggga ccca
4631 agatga ctg acacacaga ttg caaaggtcct cgg ccaggga tctcttgca

CENPN (NM_001100624)
2161 aattctggagag gtc t ggttccag tgg ctggttt ccaggga ttgattctt
3071 aaggaaagaggcttaa ctg a cgc acatttccac gtg g ccaggga ggcctc

CEP55 (NM_018131)
81 aggacccgcagccccggg gccg ggc cggt ccgg accg ccaggga gggcag

CGNL1 (NM_032866)
181 caaggcag gct cctacggt gtc agtattcg ggt ccaggga
5611 gagcatagca tcg ggagg atg ctaca tat ccaggga ctcc

CHD2 (NM_001271)
5801 acgaatgtctgatcaccgccc cgct atggg cta cca tgg ccaggga ccct

CHD9 (NM_025134)
2721 ga ccg tcctccttctaatat ttgg aagaaaataga tcaa t ccaggga cta
8531 atta gct cttaacccactattactatcta ata tactt tat ccaggga tgc

CHRNA2 (NM_000742)
2291 tgagcaggtgcctctc cgc gga gtctgg aca ctgac ccaggga ttgcagg
2621 tgaacaa tgtt taga tttgg agatgagc ccaaa gtg ccaggga gaacagc

CLDN5 (NM_003277)
1531 gctg gtctttactcca tcgg cagggc ccga gc ccaggga c

CLN8 (NM_018941)
2801 tggtgtgatctcggctcac tgc aacct ccacctccc gggtt ccaggga tt

CLPB (NM_030813)
241 ccgga cgt ggggca gcc accgggg ggc g ccaggga ggacg

CNTN2 (NM_005076)
2351 ggagcctagtgg gcc c tcc agcaaaatcc gga ccaggga a
5761 ggcagacctc ctg ggagacccacg aag ggtt ttt ag ccaggga aaactga
6451 ttatc tgct ttcattcagggcaacaaatga tac aaatg gtg ccaggga gt

CNTN6 (NM_014461)
1051 agaag gttg gacgg gagc ccgtt g ccaggga aagtcaagt

COG4 (NM_015386)
881 cca gcc aatagtggaga ccta tta tggg ccaggga gactc

COL2A1 (NM_033150)
1381 caagggagaac ctg gcc ctgct ggc cc ccaggga gcccct
1931 cgaggcgagcagggtgctc ctg ggcc atctg ggtt ccaggga cttcctgg

COL4A1 (NM_001845)
541 tgcctggttt cgctg gaaatccc ggacc acca ggctt a ccaggga tgaag
621 cccgggatgc tgttg aaaggtgaaagag gattt cccg gaatc ccaggga c
3721 caggaattc ctg gatccaaaggagagcaaggattcatg ggtcc tccg gggcc ccaggga c
4061 aggatttcaag gtc caaa aggtc ttcct ggcct ccaggga attaaaggtg

COL4A2 (NM_001846)
671 cccaggggaag gccg ggctacgatgg ctg caa cgg aac ccaggga gactc
841 tgaacctggaga gcc t ggatt ggtc ggttt ccaggga cct
901 gggacagatgg gtc c agt tgga gct ccaggga gaccagga
3081 aaggca tgcc tggactcaaa ggg agacccggg ttt ccaggga gcaaaggc
3481 attcataggaa gccg g ggtg acaaagg tgcc ccaggga ga
4111 caacatct ctg gggca cct ggtgacaaag ggg cg ccaggga tatttggcc
4451 cccgggat tgc aggaatcccccagaaga ttg ccgtc caa ccaggga cagt
4561 cc ccg gagaacca ggtttt cgt ggggct ccaggga aagct

4681 tg gcc aagaa ggt gcaccag gcc gt ccaggga gcccgggc

COL4A3 (NM_031362)
541 g gtt ctaagggtgagcag gggttt cca ggactc ccaggga
3041 aaggatt cgc aggaaatccaggtgagaaagga aac agaggc gtt ccaggga tgccaggtt
3281 gaccag gcc tccc aggt attcatg gtct ccaggga gataa
3911 caggtg cgcc t ggtcc ccct ggacc t ccaggga gtcatgt

COL4A4 (NM_000092)
1931 ggagac ctg a ttct gctcca ggaa aa ccaggga agccagg
2951 acctgggaccc ccg ggaaagaggggtc ttt cag gag tg ccaggga taaaa
3121 ccac ctg gccattcct gtg aaagagg tgc t ccaggga taccagggcaacc
4101 ccctgg gcc aagagg cctc cag gggg at ccaggga tacca
4321 ccaggtcct cgt ggaaagaaa ggtcc ccca ggacc c ccaggga gttcagg
4671 tgggccac ctg gagagaa gggttt acct ggacct ccaggga gaaaagggc

COL19A1 (NM_001858)
1821 ccctcccgggcttccag gtc caaaaggtg agg ctggt cct ccaggga aaa
2051 ctcgaggtctcc ctg ggtt gccagg aact ccaggga ctcc
3251 cagcaa tgttg gctgcccaagctta tgg gagacctggg cca ccaggga ag

CPEB1 (NM_030594)
1841 caggcaagcagc ctg caccaccc tgcc act ggcg a ccaggga gctggctt

CPLX3 (NM_001030005)
601 ggcccccatgagg gct aa gagt gtgtca actt ccaggga cccatactcca

CRB2 (NM_173689)
201 gagcccccag tgcc t gtgc ctcagacccgt gcgc t ccaggga ccgagtg

CRTC1 (NM_001098482)
991 gacgcac ctg ggca tcggt ggc gccgg ccaggga atgagc
4471 ataagaac ctg gtggac cgag gtccgag cttg ccaggga c
5611 ctgagtttt ctg tatttccaa gga gccc tccga ccaggga

CSPG4 (NM_001897)

3811 aagctggt ccg gcacaag aagat ctac gtctt ccaggga g
6011 agga gcc a gaggc agcatacc gcctc at ccaggga cccca
7051 tactgggtgtgaggcctg gcc tggg cccagat gctg atcggg ccaggga c

CTDSP2 (NM_005730)
751 gctccag tgtc tccagtacca gttct acc agatc ccaggga cctgcctgc
4641 aaa tgctg gta aat atttac att cag ccaggga agaggag

CTNNBIP1 (NM_020248)
121 cggacccgctgcccc tgccg gcccg gccg ggtcggg cggc ccaggga ccg

CTNS (NM_004937)
1661 gtacagaaagaga ccg gggtatgacca gctg aac tagc ac ccaggga ccc

CT45-2 (NM_152582)
261 gagaaggtg gctg tagatcctgaaact gtgt ttaa acgt c ccaggga

CUX1 (NM_181552)
2401 cccagga cgcc ccc ggg ctgga ccc ccaggga gcagccga
2621 ccagcctcgg gccg agc gcagtca gct ccaggga ccctcg

CYB5R2 (NM_016229)
591 gagg gcc aaggggacg cttgt tttacc atggg ccaggga atcttggaatc

CYC1 (NM_001916)
501 aggttcaagacg gcc ccaatgaagatggggaga tgt tcat gcg g ccaggga agctgttcg

DCLK1 (NM_004734)
491 aactc tgtcg ga taa cgtgaat ttg cc ccaggga gtgaga

DIO3 (NM_001362)
1821 ta gct tgagaggcatt ggcg aggttcgcag cgcc ccaggga gagagaaaa

DMD (NM_004009)
7011 aagtta ctg gtggaagagt tgc ccct gcg ccaggga attc

DMN (NM_015286)
4981 ttgaacagaagtcat tgc agt tggg gtgt tttg t ccaggga aacagttta

DMXL2 (NM_015263)
6191 aggaagacga tgctg ttg gttt agtgatgaaaagtac agat g ccaggga a

DNAH9 (NM_001372)
7631 gaagcct ctg gaaaagaaggct ggc agaaactatg gcc ct ccaggga aca
10771 ctcaggccacc ctg a tca acttcaccg tga ccaggga tgg
12241 a tgc ggtggtggcagaaaga cga aaat ttg ggcc ccaggga tggaatcgc

DOCK2 (NM_004946)
4041 atcctcag gcc caaaccagactactttg ctgttgg ata ctacgg ccaggga ttcccctcc

DOCK7 (NM_033407)
5981 gatcc cgc agaccccaaa atgct tcag atggt act ccaggga tctgtagg

DOK7 (NM_173660)
471 gcaatga tgtc c tcg tct tgg ccaggga ca
1331 ccctcacagggca gcc ccggc aaca gtgcgg ccaggga ct

DOM3Z (NM_005510)
771 ggagggctggca gctg gc agcc tccc ggtt ccaggga aca

DST (NM_015548)
9561 cattggcca gtc cttaaa ggtt ttg agct ccaggga ggat
16381 ccttaactgcat tgct at ggaa ttaatttttt ttcc ccaggga aaactag

DUOXA2 (NM_207581)
1331 ggaagggcactgagcgc tgctg gcgcgag gcctcgg acat ccgcaggc a ccaggga aagt

DYNC1H1 (NM_001376)
4801 tctcccc ttg ttatgg atgtt ctg aacat ccaggga gtac

DYNC2H1 (NM_001080463)
1711 ctcttagacca gct taaactatatgaacaggaacaat ttga tga ttgg t ccaggga tatt

4871 aca ttg atacaa gttc tga ggat ccaggga atactgaatc

DZIP1 (NM_014934)
6671 gccctagctcagg gct c tgc attcc gtg ccaggga cgcct

EDAR (NM_022336)
1331 gtgcctgc tgtcg ctgg ttcac ctgg ccaggga gaagtct

EDARADD (NM_145861)
1221 aattcacca tgtc tat tctcaagatcc atg ccaggga gct

EFCAB4A (NM_173584)
731 gaccgg gct cacac tgg cttcctca ccg ccaggga gttct
781 gggga tgtt tg tgg gggtggcg tca gc ccaggga gcgaac

EGR4 (NM_001965)
301 cccag cgctg aattgccc cgg ctgc ctg ccaggga cgctc
841 tacg cgcc ctgggagct gctttc tgt gggggc c ccaggga

EHBP1L1 (NM_001099409)
1641 caaggg gct ctcaggggagg ctgg gag tcag ga ccaggga
2151 aggga ctg gagacccaggaaacagaggtggg ggtc ataga gacc ccaggga

EIF3I (NM_003757)
621 tgttaaggagcactc ccg gcagatc aac gacatcca gtt at ccaggga ca

EIF4G3 (NM_003760)
4361 agtgggagtgga gtc caccc tgg aaaggagccaga tca ccaggga tcaca

ELFN2 (NM_052906)
671 ctgggg gtcg cccccaagat ggt ggcg gcc ccaggga gga
6001 aggagca cgcc tg ggc tgg gcc ccaggga g
6981 gaaaggaacc cgt gggaagaaggagcac gcctg ggc tgggc c ccaggga g

EMILIN2 (NM_032048)
1801 gt tgttg aagaca tttg cctgc tgaa cat ccaggga aagc

ENAH (NM_001008493)
3121 catgtcacactaatatgtagtg gcc ttttcaa ggc cta gtc ccaggga aa

ENTPD1 (NM_001776)
2011 cagctgaaata tgctg gctgga gtga ggaaaaaaa tcgt ccaggga gcat
8611 aagatca tgc atctcaaaatttaat gtacat acaa attac ccaggga ttt

EN1 (NM_001426)
2111 gaacg gcc tggc gctg cacctca tggc ccaggga ctgtac

EPAS1 (NM_001430)
141 tttttttct ttg aaaactcag aaaag tgactc ctttt ccaggga aaaag
391 ccgcccgg gccg cggggagcggacga ggg ccacagcccccca ccc g ccaggga
4471 ccccactca gccg gcagccagat ggcc ccgcaa ggcc t ccaggga tggcc

EPHA7 (NM_004440)
2141 aatt cggtg aagtctgc agt ggccgtttgaa act t ccaggga aaagagat

EP400 (NM_015409)
681 tgtt tcaggatg ggtcaggg ctca cccagatc gc ccaggga
3761 ttcctggagct ctg gaccatggtgc act tcct ggt c ccaggga tctccag

EPB41L4A (NM_022140)
3701 tctactccactaatatcaatca ctg t gtt actt aat c ccaggga gaattt

EPB41L5 (NM_020909)
3881 ggaaa gct caggaatttt aat cttgcact gtt tc ccaggga gctgtagtg

ERICH1 (NM_207332)
841 caccg gccg gggaggaaga cgtt aaa gacg ccaggga gga

ETAA1 (NM_019002)
201 tggcggcggaggaa tgc gg ctcgg tgg tcgag ccaggga g

EXOC2 (NM_018303)

2311 tcctaaatat cgcag aacatt ttg aaaagca caa ctt ccaggga atagaa

FAM107A (NM_007177)
1881 ccat cgctg gcccccacctcc tgt ggactg atg cc ccaggga

FAM150A (NM_207413)
481 aaacatttcca ccg actc tat taca ata ccaggga gtgct

FCGR2C (NM_001005411)
41 gagaaggctgtgac tgctg tgctctg ggcg ccagct cgct ccaggga gtg

FETUB (NM_014375)
1071 tttcctg tgc atct gga cctaaccacgaa tcc ccaggga gaaaccctgga

FGFR1 (NM_023108)
2121 cagacattcattgtcc ctg aatg ctc catt aat ccaggga

FGFRL1 (NM_001004356)
481 actacaccct cgtcg t gctgg atgaca ttagc ccaggga a

FLAD1 (NM_025207)
1351 ggacccctggaggaatgcttg gcc tacc tga ctgcccgt ttg cc ccaggga tcgctggtc

FOSL2 (NM_005253)
1861 caagcgctccagg gccg tgag ggca agagggggacc tgcc a ccaggga gc
2691 cattctgccc ctg gaccc ttc tctcc gga ccaggga ggcg
3371 gagtttct tgtc cagcaggg cct tgac agg aat ccaggga

FOXP2 (NM_014491)
721 accaacg gcc cc agtc acccc gatt ac ccaggga ccctca

FUK (NM_145059)
561 t ccg gggagcca gag tgatcgcc ctc ccaggga gcccggc
861 ttctcca ctg catg gctgagaa cgtg a ccaggga ggactt

FUT4 (NM_002033)

5161 attataaga tgtt ctaaat ttt caaggatct aaa ccaggga ttggcaaac

FYN (NM_002037)
251 gcgccgcaccgc ccg gcggc cgc cgccc gcg ccaggga gg

GALNT4 (NM_003774)
301 cttttca tgcc tc cgcagg agccgg ccgtg ccaggga gct

GALNT7 (NM_017423)
421 tcacatttaa gcc tcagacattcacctaccatgatcct gtg ctt cgc ccaggga tcctcg

GATA5 (NM_080473)
1781 ggtgcagagaacttttcc cgc aaca ggt gcagg act g ccaggga tcggag

GCET2 (NM_001008756)
1841 tttccttag tgtt cctcaaa tta tatctatcctc taa a ccaggga tcagc

GDE1 (NM_016641)
2521 atgaccccaaac tgct ct ttc caattt gaa ctt ccaggga ttttattgtg

GLP-1 (NM_001103167)
391 ggccatgcaattcca tgtc tct ggggctg atgtagg cactcc ccaggga t

GMEB2 (NM_012384)
61 ttgagttcag gcc aggagagacggaagaactg gcaagc cggga gcctgc gt ccaggga ag

GNB1L (NM_053004)
611 agcca cgctg g acg cttgc cgt g ccaggga

GPRASP2 (NM_001004051)
1401 tacag cgtc ttctttctggactggagaagagacaagt gtca gatca tggc ccaggga aga

GPR22 (NM_005295)
481 aaga tgtt cttgtaacacgactttaagaca ttaa ggag ttaa aa ccaggga ata

GPR44 (NM_004778)

2441 tggacttggggtcagagac tgctg tgtttga gct ctgc agc ccaggga cc

GPR97 (NM_170776)
541 c cgtc acca ttctggacat tggt ccaggga
2291 agacccccgtgttcagag tgctg gca gccctgcacg tgt ccaggga cact

GPR103 (NM_198179)
761 gc tgttg tgacagaaatcctcactatgacctgcat tgct gtggaaa ggca ccaggga ctt

GPR110 (NM_025048)
151 caccagtcacagactatgcac ccg actg ctgctgtt cagt ccaggga aaa

GPR114 (NM_153837)
2341 ctt tgtc ctggtacc tgggc cca gctcg ccaggga tgtgg

G6PC2 (NM_021176)
1761 cacatgctttttgt ttg tatgt ttc cttttta gaa ccaggga cttgctct

HDAC4 (NM_006037)
4121 cacgcctg cgtc ccaccgtggg gctct ctt ggagc ac ccaggga caccca
6201 ccctggatt ttg gag cctgtg gctg caagg aac ccaggga

HIST1H4D (NM_003539)
251 ccatggacgtgg tgt a cgc gctcaa gcg ccaggga cgcac

HIST1H4G (NM_003547)
251 ccatg gccg tggtcta cgt gctcaa acg ccaggga agaac

HIVEP2 (NM_006734)
3921 cctg tgtcg ggagcagcagcctcc acggt atc accgt ccaggga gaggaa
4111 cgcctgctg tgctg cctcc tcttc agcaa gagga c ccaggga agcaggtg
6821 ca tgct accttc agag ccaagctc ctct c ccaggga cttc

HIVEP3 (NM_024503)
771 cag cgt ggcatc tcttg tgc cagga ccaggga tgactggg
7141 gccg agtcaccacca cggt cagc gccg ccaggga agtggg

HMGCL (NM_000191)
641 gatctcc ctg ggggacaccatt ggtg tggg cacc ccaggga tcatgaaag

HNF4G (NM_004133)
2701 tgactg gct tagcagattct ttg aaatgtag cag c ccaggga tgtaccaa

HSF4 (NM_001538)
1831 aggggcccaggaa tgcc caacag cct gaaccag ggg atc ccaggga gata

HSPA12A (NM_025015)
431 ggaagttccaca gct t cgg gtatg ccg ccaggga ctttta

HS6ST1 (NM_004807)
2021 tcgggccaactggca gcc caggag tgggg agg ctttg g ccaggga tgctg

H2AFX (NM_002105)
351 agctcaacaagc tgctg g gcgg cgtgacga tcgc ccaggga ggcgtcctg

IGF1R (NM_000875)
561 gggaataa gcc cccaaagga atgtgg ggac ctgtgt ccaggga ccatgga
5121 ctcccccgcc cgc ccccaaggacacagatgg gaa gggg ttt ccaggga ct

IGSF3 (NM_001542)
801 cacggagg gct cccacatcactatc tggtgc aatgtga gtggcta ccaggga ccttctga

IL2RA (NM_000417)
571 agaatttatcattt cgt ggtggggcag atggtt tattatc agtgcgt ccaggga tacagg

IL4I1 (NM_172374)
1431 ggcttg tgctg ttgaacgcgcc cgt ggtggcg atg ac ccaggga ccgcac

INCA1 (NM_213726)
1111 agaaaccaccaa tgccg aga ggc tagtggt gcc ccaggga

INTS10 (NM_018142)

1331 agccgaact tgct aactccactgaagtg ttag aaagctttaaa ttgg ccaggga gagctg

IQCE (NM_152558)
41 gcggcctggt tgcc atggcagcggg gtcg cgggc cggc g ccaggga aggc
2531 cacat ctg c cgt gaactcgcag atg ccaggga gccctgta
6441 cccgtggc tcg ga ttct ctg agga ccaggga gttgacaca
5441 attcc cgctg ctcacagacctcagcagaggcacc agct ggag ggct ccaggga catcctt

IQGAP3 (NM_178229)
1821 tcctggagctg tgctg tggctt gagga gatccg ccaggga

ITGA7 (NM_002206)
441 tgcc cgttg agcctggaggagac tgactgctac aga gtggacatcg a ccaggga

KCNK17 (NM_031460)
901 catg gctg gccttgatcatcaaactcat cctctcc cagct ggagacg ccaggga gggtat

KCNN2 (NM_021614)
991 gatcatcctgctcggt ctg atcat cgtg tac cacg ccaggga aatacagt

KIAA0195 (NM_014738)
4551 cagtcctg gct cttccc tgg gcc tca ccaggga cactctt

KIAA0226 (NM_014687)
991 gatcaaaccatccaa gcc cccccagttt cagt ctctgc actag ccaggga

KIAA0355 (NM_014686)
1991 gcaggc tgct ata atg gaatca cat ccaggga tgattttc

KIAA0409 (NM_015324)
941 gtgaagaagtggccactgca gcc ag tgg accgca tcg ccaggga tcttcg

KIAA0644 (NM_014817)
1 ac tcg gc tggg agaa tccg cg ccaggga at

KIAA0664 (NM_015229)

4031 ggacagcca gcc agc ggc ccc gtc a ccaggga gcccgact

KIAA1026 (NM_001018001)
2201 tacca tgctg ctacccaactgtgcaaagta gtttagg gtgg ccagaac ccaggga ccatt

KIAA1191 (NM_020444)
1271 tcacacccactg gct tct agag ccct cttt ccaggga ttc

KIAA1210 (NM_020721)
51 ccttcctg gccg gcacccctacct ggctc acct gggcc ccaggga ccgag

KIAA1217 (NM_001098500)
1581 atggttgttc ctg gca atg ccac cat cc ccaggga cagaa

KIAA1644 (NM_001099294)
781 cccggtgcagcct ccg agaagaacaa cttct acag agatg ccaggga cag

KIAA1946 (NM_177454)
1871 cccaaaa tgcc aattcattct cat gcacagcccccag atg ccaggga aga

KIAA1967 (NM_021174)
91 tgggagcaggagggtggcg cgc ag ccggtt tcgcgttt ggtcgg ccaggga gctgccttc

KIF1B (NM_015074)
7091 aaacaaaacacaata tgcct aggggc acg gatgaa cgt ccaggga gcccg
7241 ctccat ctg gt gtgg aaaca ctgc ccaggga gaaaggagg

KIF13A (NM_001105568)
4331 accaagatgtagca tgtt a tgg aact tta c ccaggga ttc

KIF13B (NM_015254)
3961 cttttgaaat tgtc tccaa tattc caga ggatg c ccaggga gtgg

KIRREL3 (NM_032531)
2461 g gtc agggc acg ttctggt tgt ccaggga c

KLHDC6 (NM_207335)
2381 gatgaccaa cgcc tgaaagaatgaggag ttg gacctggtctg taa g ccaggga attgtct

KLHL23 (NM_144711)
1221 ttgacaaatgt ttg gattcagggagcagaa ata ccagat tat a ccaggga

KNDC1 (NM_152643)
1231 agaccctcgaga tgct agc ggt gaagcccag act c ccaggga cgatgaga
6041 gagag tgcc cccacc cat ctc atg cc ccaggga ccgccac

KNG1 (NM_001102416)
1141 aggtggtg gctg gcaagaaatatt ttat tgacttc gtgg ccaggga aacc

KRT38 (NM_006771)
1891 gtatag gcc tgtcca aaggcta tgagata ccaggga cagt

LARP1 (NM_015315)
71 cagagctggatttccaagag gct cccatacct agctgc cctg gcagactc ccaggga gga

LATS1 (NM_004690)
1001 ggctgcagcagc tgcc agaccta ttaatg ccag catgaa a ccaggga atg

LCOR (NM_032440)
1771 ccagatgttt ctg taaa gatt gaatta gatc c ccaggga gaggcagcaca

LGR6 (NM_001017404)
31 agaacctctcccgg gctg gga gtgcac ggcgcg gtgcgc ccaggga cctc

LHFPL4 (NM_198560)
3731 acctggcca gctg ccatcttg gca gagccaggggggaga tgc a ccaggga g

LIPK (NM_001080518)
171 tatcc ttg gaatttataggattcca cat ggaagagg atg c ccaggga gga

LNX2 (NM_153371)
1531 cag gcc agtggagagagagtgaa ttta acaattgc taga ccaggga aacc

LOC646892 (NM_001101404)
1051 tatga gtt catcgggacagaagg cctc ctgcaa gagg ccaggga cacacc

LRRK1 (NM_024652)
6251 aacacagagtgg tgcc tggc cgtct ggaggggctg gggcg ccaggga gtt

LSR (NM_205835)
1411 cccccgga gtc ccagggga tggg accaggag cccg ccaggga gcaggcag

LTBP1 (NM_206943)
601 tgtctccg gcc acaactc tgt gtgt gta aa ccaggga cca

LYNX1 (NM_177457)
2091 cagggtggaa gcc ccagct gggt gtgtgg actc ccaggga ccccacccca

LYPLA2 (NM_007260)
51 agaggagaat cgcc caagc ggcct cgg aagtc ccaggga gtggaggcccc

LYPLA3 (NM_012320)
1701 ctatgagggatgttactgg gctg tg gtcct gtacccag aggtc ccaggga

MAPK1 (NM_002745)
891 tctggcagaaa tgct ttctaac agg cccatc ttt ccaggga agcattatc

MAP1A (NM_002373)
2141 attccctctagacac tgc agaggagggacccccaa gta cagc tat ccaggga acaccacc
7741 gtgcctctc ctg a ggtc gaagctg ggcc ccaggga tgtgc
9211 ccc tcg taga gggaga ttata tcccc aact ccaggga cct

MAP2 (NM_002374)
3991 tgctcttc cgct cagacacccttcagataactg acctggg tgt ctcaggt g ccaggga gg
4021 ctgac ctg gg tgtc tca ggtg ccaggga gg

MAP3K14 (NM_003954)
3001 ctcggtgcacga tgctg ccctgaaacaca ggc tcagcc gtt c ccaggga t

MAP4 (NM_030885)
2401 gtacagttatttattaa gttg attt tgggg tttc tttcg ccaggga tctt

MASP1 (NM_001879)
2181 gctta tgcc c cgct gaagaagaa agtg a ccaggga catga

MCTP1 (NM_001002796)
461 ttt tgct ctcagtcatcc ttaccct aaaga aggagag t ccaggga tgtg

MDGA1 (NM_153487)
4121 ccccgccagctgggg tgcc caggggca ggg ccgg ccc g ccaggga ggggg

MED12 (NM_005120)
4481 gtggcccccctcat tgct aa actg cccacct cagt ccaggga catgtgtt

MEGF8 (NM_001410)
1261 accagggg gctg ggtggtgg cac aacgtga gtg ccaggga ccctgccttc

METT11D1 (NM_022734)
1081 tggagaatggaacaaaa gctg ggcacag ccttc tcat ggatg ccaggga t

MKL2 (NM_014048)
7471 agcaggtgcg tgtt ttttcc acatt cgccctttcttgc agtat ccaggga aacacatcat

MPG (NM_002434)
461 cgtccgatgca gcc ca ggca cct tgcc ccaggga gcgctg

MRAS (NM_012219)
941 aggtcgatttga tgc act tga ggaaga tca ccaggga gcaaggaaaagaa

MRPL43 (NM_032112)
171 cagccgcgacgg cgcc t cgtc tcgc ggcg ccaggga gttc

MRPS26 (NM_030811)
651 actcctaggg gcc ca gta aggacag tgc ccg ccaggga cc

MSC (NM_005098)
211 cttggag cgct ctc tgg cctccgcct ccg ccaggga gcgg

MST1R (NM_002447)
2741 ttcctgaata tgt ggtc cga gacc c ccaggga tgggtggc

MUPCDH (NM_021924)
291 cggagcc ttgt ccaccccc tttg cattt cgga t ccaggga

MVD (NM_002461)
1551 tggggaggtg gtc cc tcc agc gga ccaggga aggggtcac

MXD4 (NM_006454)
121 tgctg ccctt cgacgg cga cttcg ccaggga gaaaacaaa

MYBPC3 (NM_000256)
241 acggcata cgctg acagtgcgggaagtg ggc cct gcc ga ccaggga tctt

MYL9 (NM_006097)
31 cacgcacccagcga gcc caa gcg ccttctc cgc a ccaggga agccccacc

MYO1E (NM_004998)
3411 gagc ctg gatt tcc tcaaggtccc gga ccaggga gctgca

MYO18B (NM_032608)
1051 gccccaa gcc caagg gccc ggcgagg gggt gcga ccaggga aagcagaga

MYPN (NM_032578)
851 gaaagatctt ctg ttcccatccc tatcc ctgc ggata ccaggga taatga

MYST2 (NM_007067)
2531 ggctaaccaga gcc aaatactttt gaa gagt ttc ccaggga ctagtcatg

NAG (NM_015909)
921 aca ctg ggattattaaggat gttaag tgtcaagt tttacagt cg ccaggga

NBL1 (NM_182744)
1 gatgcaggctgggtgtggaggg tgc ga gta ggctggag tac a ccaggga gttcccagcag

NBPF1 (NM_017940)
3031 gatgttcaagttgaggtg gctg agaaagt gcaga aatcg tctgc cc ccaggga gatgcag

NBPF20 (NM_001037675)
601 a gttg aggaggatgagaaa gtactgg aatca cctgc cc ccaggga ggtgc

NCBP1 (NM_002486)
1451 cttg tgctg cac agtt agtg agct at ccaggga agaacaa

NECAB3 (NM_031232)
1381 ccagggct tgtc tcctcctggacttgggc ctggtgg aagggc tctcag c ccaggga tcag

NEO1 (NM_002499)
4741 ccctt tgcct gctgatattctgcaggactgggcac catggg ccaaaat tttgtg t ccaggga

NFKBIB (NM_002503)
491 gccc tgct tcagccc cgcc ccc ggcg cc ccaggga agccc

NGB (NM_021257)
191 actg gccg cg cgc cttttctctccc gcg ccaggga aggag

NLGN4Y (NM_014893)
451 cggatgtgga tgc a gatttgaac cat gttgcgtc c ccaggga ctgctatg

NOVA1 (NM_006489)
591 ttacccaggtacta ctg ag cgag tgtg cttg at ccaggga

NPC1L1 (NM_013389)
961 ttgtgggatt ccg tg tgg ccc ccg ccaggga caaaagcaa
2481 gtctgc tgctg tgtcaagccccaggag ctg cccccgcc tgg ccaggga ga

NPW (NM_001099456)

581 cgcgcg gccg ccgg gccc ctgg ccaggga c

NRF1 (NM_005011)
3321 taattattatttttat cgtc att gtg aagt tgt ccaggga ctttaaagt

NRXN3 (NM_004796)
1511 catgtcccagcgagcttatgg gctg ct ggt ggctacg acc t ccaggga ct

NTSR1 (NM_002531)
2961 ttcaagggatttcc ctg tctcaga gcag cct ttgc c ccaggga aatgggc

OPN3 (NM_014322)
2021 ttt tgttg tttctctaaaatttactgttc tttgt cgatgctat ataag ccaggga gttc

OR11G2 (NM_001005503)
141 cactggcttcatcctc ctg ggc ttccctt gcc ccaggga g

OTX1 (NM_014562)
2001 gcgggaa cgctg tacat agt caggtcc gtt ccaggga cca

PALM (NM_002579)
1231 gtgc tgccg agcctcccacgg agg ccg cct ccaggga aga

PAPLN (NM_173462)
3021 cg tgctg tctgaggctgagctgagcc gct tccctc agc ccaggga cccag
3571 gatggca cgctg ctca ttt acaacttgc ggg ccaggga tg

PAPOLB (NM_020144)
171 gcggg cgctg aaagatgatgcc gtttc cggt gacaac ccaggga ccaccg

PAPPA (NM_002581)
5331 acagtcagc tgct caac gga atggcctc tcc aca ccaggga tccttagca

PBX2 (NM_002586)
1491 ag gctg tgacccctcttcagtgaca tccc caacgga ggga ccaggga gt

PCDHB16 (NM_020957)
1921 cctcttg gct ccc tggttgc cacc gtctccg ccaggga tt

PCDHGC5 (NM_032407)
931 gaggagtca cgtt tctatgaaat tcatg caagagcc cgtga ccaggga ca

PCGF2 (NM_007144)
621 tgatgatgagat tgtc agcctctc catc gaattctacgaa ggtg ccaggga ccgggacga

PDCD4 (NM_145341)
2221 aaaaatta gtc at gaga cttattca tctt t ccaggga aca

PDCL (NM_005388)
631 aaaagcattgtcatcatg gtt catatttatgag gat ggc att ccaggga c

PDE4D (NM_001104631)
571 gcccggcctgaagaaatccagga tgtc ct ggc cctcctc gtt ccaggga c

PDE4DIP (NM_014644)
4871 gacaga gct tc cggg agcaa ctcg c ccaggga agccagct

PDE6B (NM_000283)
1 tgcg tgcc t ggagc agca gcgtct ccaggga caggcagcc

PGBD5 (NM_024554)
2881 cgtgtgtcccccaggcagat ctg ggcactttcccaaccc aggt ttatgc gtct ccaggga

PGPEP1 (NM_017712)
931 cctgcat ctg ggga cac agctgcc gtg a ccaggga ggcca

PHF10 (NM_018288)
951 cctcctgaca gcc ag gagg act cttt ccaggga agacaga

PHF14 (NM_014660)
151 ggggccgccgggttgactg cgctg cctggg ccggagg tct tctccgg ccaggga gcgctg

PKNOX2 (NM_022062)
1451 catcgctgcggggg tgctg cagca gca gggcgg tgc c ccaggga caaacc

PLCE1 (NM_016341)
551 taatca gtc attttattaaaacct tga catga tca ccaggga ggaaaaat

PLCG2 (NM_002661)
331 agatcgagg gct tcttggatatca tgga aataaaagaaa tccg c ccaggga agaactcca

PLCH2 (NM_014638)
751 cgaggacagcctg gct cgc cgcca gcg ca ccaggga ccag

PLCXD1 (NM_018390)
161 ctgcaga ccg agaggatc tcgt cctttctt gcgg c ccaggga gaccaggc

PLD2 (NM_002663)
61 ggctcgggaacccc cgc gggcg ctggc tcc gtctg ccaggga tgacggcg

PLXNA1 (NM_032242)
1791 caccctc cgcc cgggaggtggcg ccc atcacgcg ggg ccaggga gaccag
3591 tcacggtgcgggcaggtg gct t cga gttc tcg ccaggga c

PMEPA1 (NM_199170)
1921 gccg cagc ggct ggtgcccg ggct a ccaggga catgccag

PNPLA6 (NM_006702)
1241 aatg gtc agcacctca gct acagacg agc ccaggga gacc
1711 gcagagtct tgctg caccacgccaaagc tgg caccatcattgc ccg ccaggga gaccagg

PNPO (NM_018129)
2881 gctgaactgtgtt tgtt ctcc ttgagc acatgccc gctaa ccaggga cag

PPAN (NM_020230)
1391 ggt cgcc tttgtgaccagaagtttcccaa gac caaggacaa gtc ccaggga
1611 gga gttg tgtccccagcccttccact ccagt aaagaactga attgg ccaggga

PPARD (NM_006238)
2161 acactaa gct c tctg ggcc tggg tt ccaggga aggctaag

PPFIA4 (NM_015053)
51 taggcataggggta tgcc tggc ttga att ttaa ccaggga

PPM1A (NM_021003)
271 cccggctgc cgccg cc gccg cct cggc cga ccaggga cct

PPM1F (NM_014634)
211 c tgctg aacccagaggaccctct gcc atggaa ggc c ccaggga cggtgct
371 a gctg ctacagacagacctttccg aatt cagga agtt gc ccaggga ggaa
4141 ttccagccaca gtc ccatg agg gcc cct ag ccaggga cac

PPM1H (NM_020700)
5841 tttactcctgttt tgcc cccagttttcttt gttttt tttct ggagac ccaggga ggccca

PPP4R1 (NM_005134)
3031 ctgcggacttcattgcagg tgc aa gttg cctacacc caat a ccaggga tt

PRICKLE2 (NM_198859)
421 ctgcctgaggtcc tgct tgc gtt cttagaagtca gat ccaggga gaaagt

PRIC285 (NM_033405)
2511 atca ctg at gtg gccagcttcg tgc ccaggga cggggtgc

PRKCA (NM_002737)
7101 aacattcc tgtc tcctccc atttg ggctgatgcag cagat ccaggga atg

PRKDC (NM_001081640)
5191 cgtgttctggagcagctcat cgttg ctc act tccccatgc agt ccaggga

PRPF40A (NM_017892)
5951 ga tgct atgtaagagaaa actat ttgtaatcac atagt ac ccaggga gga

PTPRB (NM_002837)

3971 cagaagga cgc attgtgt atgg tct tcgt ccaggga gatc

PTPRN2 (NM_002847)
2481 tcacgac ccg aggaacccc gcg tacat cgc cac ccaggga

PTPRT (NM_133170)
7151 gggct ctg ga gct gtccaga agt ccaggga
10041 atgtcaacag gttg tgct ggg ccagga tcc ccaggga aaa

PVRL1 (NM_002855)
5301 gcttcggcgtct tgtcg cccttt ccg cctt tgg t ccaggga cagcccagc

PXDN (NM_012293)
541 gctcgagag gct atttttgcataacaacc ggat tacac attt agtt ccaggga catttaa
721 g gcc atc tgtg aatatcccaga cgca t ccaggga cgctca

RAB11FIP5 (NM_015470)
1011 ccctgtcctca gcc agc ggg agcttgg cct a ccaggga cc
2431 tcccacctgcc tgtt ttcatatccctca gctt tgg gggc c ccaggga att

RALGDS (NM_006266)
1511 gaagaaga cgt ggg aagacg ttt ccaggga

RANBP10 (NM_020850)
1 ggac tcg gactcg gtt tccctgc ggc ccaggga ggctcac

RAPGEF4 (NM_007023)
551 ccccgcca tgc aac catc gtt a ccaggga g

RAP2B (NM_002886)
211 gccgccgcggac tgctg cg ggg cccgga ccc gcacc ccaggga tacgctg

RASAL1 (NM_004658)
691 gctgcctt cgctg cca tgtg ctt cacg ccaggga cctggc

RASL12 (NM_016563)

81 agggcgcgga cgccg acgggtccgg ggc cgcgc gct ccaggga agcctgc

RBM6 (NM_005777)
1661 ga tgcc atcggatgca tgg agg cca a ccaggga actctaa

RBM46 (NM_144979)
441 cccaatta gcc atcagaattcttaat aatt atga aatt cga ccaggga ag

RELL1 (NM_001085399)
511 aacagcctgtatgatcctgaaa gcc cc gtg acccccag cac a ccaggga g

RGN (NM_004683)
181 gggctggctggtg cgcc ctc tgca aagcc tgcg ccaggga ggaggcaggc

RGS1 (NM_002922)
1201 actgtgtatttaacttaa gct a ttg ctct taa aa ccaggga gtcagaata

RILP (NM_031430)
921 cgaatgggcgac cgcc ggcg cagg cgcc ccaggga accct

RNFT2 (NM_032814)
481 gcggcggggacg tgtt catccag atg cccg cgt ccaggga

RNF4 (NM_002938)
601 gaca gctg tgtggtgagcagtgacg atga ggag ttgt ccaggga cagaga

RNF32 (NM_030936)
1331 gaactgcatgagttctgg gtt aagtactac aatg taatc tgtt tc ccaggga aataagct

RNF141 (NM_016422)
3191 tgaaaagtgaacaga gtt accttaca gag ttttggatc ttt ccaggga ta

RNF148 (NM_198085)
51 actaaatattaaaata ctg gaaggag caag atagc tttg at ccaggga ga

RNF212 (NM_194439)

291 ttcttcatgagcatagacagt ctg t gta agaag tac t ccaggga aacctc

ROR2 (NM_004560)
61 acgaggtcc tcg aagtggacc cgttt gcg aagcg ccaggga gaaggagga

RPAIN (NM_001033002)
481 cgcggtctgcac ccg gaaagtacacctcccag gggt ctgtgcgc gcct ccaggga acggg

RPUSD2 (NM_152260)
281 caggc ccg ggcaagcataagaagcg gcg ggg cgc aa ccaggga gcgtgtc

SAGE1 (NM_018666)
581 ggctgca gctg g tattc catccat gagta ccaggga tctg
861 atgacagaca ctg g tattt cacccat gagta ccaggga tc
1431 gga gctg g tattc cacccat gagta ccaggga tcagtatg
1851 acagta gctg g tattc cggccat gagta ccaggga tcagt
1991 ggcagcaa ctg g tgttt catccat gagta ccaggga tcag

SCN3B (NM_001040151)
2741 tgtt tcattttaattaagtgacaggtccaa gtgtga catcct tcagcac ccaggga caagaga

SCRT2 (NM_033129)
2881 gtttcc tgtt tattcttcttcccgagg gacc cca ggtt ccaggga ccgac

SDK1 (NM_152744)
9861 tcactcaa tgctg ta gcc aaaaaacgagg ggc c ccaggga

SEMA5A (NM_003966)
6981 acactacacaaca tgt g ggct tcctgt agct ccaggga ca

SEPW1 (NM_003009)
441 caaagc cgcc ttggctcagggcta atgcgcc ctgaa ggcagagt ccaggga ccttgaccc

SEZ6 (NM_178860)
3001 cgatggtgt tgttg gtaggaggtgtatacttcta ctt ctcc agg ct ccaggga aaaagct

SHROOM3 (NM_020859)
8521 ctgcatgactcca ttg atacaaataagac agc accttg gtt tc ccaggga

SH3BP5 (NM_004844)
291 gctg gaggaggaagaagagg tgg atcc ccg gat ccaggga

SIPA1L2 (NM_020808)
3621 caccagggctcag gcc ctttg gaatgtga cggag ccaggga gagggaaga

SLC1A4 (NM_003038)
1991 ttcggcttgatccatgtccagg tgc aac tgt gtgt aca ccaggga tctgt

SLC4A11 (NM_032034)
491 tggataacg tgctg cgga ccatgcttcgccgc ttcg ccaggga ccctgac

SLC26A11 (NM_173626)
2181 gctggcctg gctg agaaacc cct gagc agg taac ccaggga agagaagga

SLC29A1 (NM_001078177)
161 gtgcgga gccg cgcag cacgtg gcgcg cacgg g ccaggga

SLC29A4 (NM_153247)
421 gcttcatcacgga cgt ggactacc tgc atcacaa gta c ccaggga cctcc

SLC29A2 (NM_001532)
1971 ggccaggag cgcc tcatcttcccaggcctcagc cacc cagggtaaaa ggtg ccaggga ag

SLC33A1 (NM_004733)
961 gacattgccgtcgatg gttg ggcgt taac tat gtta t ccaggga aaatgt

SLC35A2 (NM_001042498)
2031 gggccagcttcc ttg ataccttgaagat gggctt cttgt gagtcc ccaggga gaaaggga

SLC39A4 (NM_130849)
891 gtg tgt ggga cac ggtatgcctga gtg ccaggga cgtgat

SLC39A7 (NM_006979)
1881 aggggtg cgt agagg ttggg ggc cctgg ccaggga catct

SLC41A1 (NM_173854)
911 gaacgggactggcccttc tgcc tctccct gct cttcagat ggc ccaggga

SLC45A4 (NM_001080431)
1631 cacggtgcgcc tgctg tggctctc cat gctgaag atg c ccaggga gctga

SLC46A1 (NM_080669)
4731 cctaagaaga gcc cag gttg tct cagc ccaggga gactgg

SLC47A2 (NM_001099646)
2111 gaagacatca gcc a act gcacgagtcag agt ccaggga tt

SLITRK1 (NM_052910)
3481 acaaaaag tgctg catggct cgcatgg aat ccacgcg ct ccaggga ctct

SMARCD2 (NM_001098426)
511 cgggccccgcgg cgc agtaccagc gacct ggc atgtc a ccaggga accgg

SMTNL1 (NM_001105565)
1 atgctcccgggggca gttg gagagtcagattg ctc agca gag g ccaggga gaagatggag

SMURF2 (NM_022739)
1681 cc ttg acta tgg aggcg ttg ccaggga atg

SNRP70 (NM_003089)
1231 gcgctcacgga gtcg cgacaaggagg agcg gagg cgct ccaggga gcgga

SOX30 (NM_007017)
1531 cacagagaggaatttc ctg gt tggg tttatcagc ctcg t ccaggga agcg

SPEF1 (NM_015417)
591 atcccagaaggc ccg aggtgaa ggt gtcccgg acc c ccaggga gggggtc

SPINT1 (NM_181642)
571 tacgagcagaact cgt gtgc aagttc gcgc ccaggga gg

SPTAN1 (NM_003127)
3611 ggtgcag gctg tgcaacaacaggaagt gtat ggcatg atgc ccaggga tg

SRD5A3 (NM_024592)
421 atggtttgctcagaattc tcg gggc ggc aca gtt ccaggga ggggagctg

SREBF2 (NM_004599)
2791 tcggtggtgga cgtc tgcaatcactgt ggcca tcagc tggct ccaggga g

SSBP4 (NM_032627)
1411 cggc cgccg ggaccttcctgcacccgttcc cgag cgaaagcta ctcg ccaggga tgacca

SSX4B (NM_001040612)
51 ccag tgcc atgaacggagacgacg cctt tgc aagg agac ccaggga tgat

SSX9 (NM_174962)
361 aatcaggttgaa cgtt ctcagatga ctt tcggc agg ct ccaggga atctt

SS18 (NM_001007559)
1051 ggaaattcacagtatg gcc aaca gca aga tgc ata ccaggga ccacctcc

STARD7 (NM_020151)
2121 cttgaactc ctg gg cca tgcc tgg ccaggga catgaattt

STBD1 (NM_003943)
781 ggcagtcttaag gct ccagtg tta aacc taa a ccaggga a

STRN3 (NM_001083893)
1221 cccaac tgctg agg tttg ggatg taga ccaggga ctaata

STS (NM_000351)
1231 tgagattg gct aatgataccctcatctac ttc acatc gga ccaggga gca

STYXL1 (NM_016086)
801 tggatgcatttca gcc ataccc cattg aaat cgtg ccaggga aggtcttc

ST7 (NM_021908)
2351 gtcacttccc tgct ta aattt ctccagt ggatt c ccaggga cttcaggat

SULT1A2 (NM_177528)
581 tgcggg tgcc cttc ctt gagttca aag tc ccaggga ttcc

SYNE1 (NM_033071)
24381 ctgacgcagctggaa ctg atcaacaagcagta ccg ccgcc tgg ccaggga

TAF1C (NM_005679)
1021 cagggacctgt ccg gcaa gtggt gacatgc accgt ccaggga gaaagtaa

TAF6 (NM_005641)
391 ggtac cgccg ggtattgaa ggattc gtag gagttc g ccaggga agtggga

TAF8 (NM_138572)
1661 ctt cgtt cattatcacaggatttgattc ctttga aac tcaagag a ccaggga aagtgaca

TAGLN3 (NM_001008272)
851 atcggagag gct tttc cgagg agcag cttcg ccaggga ca

TAPBP (NM_172209)
281 gcctggccaagagac ccg g tgca ctgctgt tgcg ccaggga ccgggggaa

TAPBPL (NM_018009)
1001 ctggagcc tgc acaa ctg ggca tgg ccaggga tgcctccc

TARBP2 (NM_004178)
1021 cggccactgtg tgtc a tggct ctgc aacca ccaggga ggc

TBX1 (NM_080646)
111 gccaggatcc ccg gcagg gatg cacttcagcac cgtc a ccaggga catgg

TERT (NM_198255)
1141 ggagaccatcttt ctg ggt tccag gcc ctgga tg ccaggga ctccccgca
3461 cagccctgtca cgccg ggc tctac gtc ccaggga gggagg

TET3 (NM_144993)
4951 aaggtcactg gcc cctacagc cgct ggatcta ggtg ccaggga gccagcg

TFAP2A (NM_003220)
1111 accc tgct cacatcactagtaga ggg agaagctgtcca cct ag ccaggga

TGM2 (NM_198951)
441 tgtatcgcctcagc ctg ga ggcc tccact ggct a ccaggga tccagcttt

TGM5 (NM_004245)
971 catgtcctggct cgtc cagggagg gaag gagcagaag cttc a ccagga ca

TIGD7 (NM_033208)
1341 gtcaa tgcc agaaaattctcaggcaagt agg aaagatatctg cct a ccaggga agaaaat

TLE3 (NM_005078)
801 caggcattga cgc gct cgacgc agc ccaggga aggcggcc

TMEM16K (NM_018075)
2491 atagcag tgtc actcccaacc cca cca tgg c ccaggga

TMEM45B (NM_138788)
51 caggtgtc ctg atggcaaatttcaa gggc cacgc gctt ccaggga gtttc

TMEM59L (NM_012109)
541 ttgttttccaccctc tgc aatgacc ttg tcaact cag c ccaggga tttgt

TMEM86A (NM_153347)
3341 agagcaaaggaagtatt cgtt ata tta atgatgc tgg cga ccaggga aat

TMEM95 (NM_198154)
291 gagttggct tcg aaagaccaa gctc cct gagt aca ccaggga agctctct

TMEM181 (NM_020823)
1181 cctgctgctcttctg gctg tgcgt gtac cacgggattc gtgt ccaggga g

TMEM184A (NM_001097620)
2711 gtgcaagtggcc tcg gaaatc gcggccgc aagaaca gtagccgc ccaggga ctaaggggg

TMEM195 (NM_001004320)
181 cagaa gcc cagca ggat gtttca gttt c ccaggga tttcg

TMPRSS13 (NM_001077263)
2791 acaga tgctg ttcccgcgta ggctc ccag gggcc ccaggga ggtccaggt

TM2D2 (NM_001024380)
891 aactggtgcac tgttt actaagaagagct gcca tca tggc ccaggga ggc

TNC (NM_002160)
5341 agtctga gcc actggaaataaccc tactt gcccccg aacgta ccaggga cataac

TNFAIP8 (NM_001077654)
301 gg tgctg gatga gctc taca gagt ga ccaggga gtacacc

TNFRSF11A (NM_003839)
1941 agcaccgca gcc tct gcc ccagcccc ggc cac ccaggga t

TNN (NM_022093)
2041 tgcag gccg ccattgacaagtacgtggtgcgctacac ctct gctggtgg agag a ccaggga
2321 caggtatgtggtg cgct acac ctctgcc aaggac ggagag a ccaggga ggttccggtggg
2591 gtatgtgg tgc actacacg tctgcc aac ggaga ga ccaggga ggttccag
2861 ggtg cgct acac ctct gctgacgg agag a ccaggga ggtt

TNS4 (NM_032865)
1411 gccagaccctgtcaga tgcc ccctttaccacat gccc aga gggt cccg ccaggga catgc

TPPP (NM_007030)
4991 cgccccacttgcc tgttg gtgccac tcc atccc ggg cc ccaggga tgcca

TREH (NM_007180)
241 gtcctgcagaccttcactga gctg tccag gga ccacaatcacagca tcc ccaggga gcag

TROAP (NM_005480)
471 caaaatgtggggcctgg gcc ccct gcc cagacaga ggc t ccaggga ccat

TRPC1 (NM_003304)
3681 tggtcttacgccacaggg tgc aggtaaccc ttggt ctgtaagcacc accga t ccaggga t

TRPM1 (NM_002420)
3971 gttattat ttg ag gat acatctctctccac gtc a ccaggga caggagtca

TSGA10 (NM_025244)
1611 aatg cgtcg gcaattggatgagacaaatga tgagctg gcc cagatcg ccaggga aagaga

TSPAN9 (NM_006675)
1001 ccctgcctcagcc tcg g acttc tcagtgggt ggagt g ccaggga ggagga

TSPAN18 (NM_130783)
2641 ttcca gcc tcccctca ggct ttcc agtc a ccaggga cact

TTLL8 (NM_001080447)
1061 tggaggagatcctggagctggca gctg c aga ccaccctc ttt ccaggga c

TTLL12 (NM_015140)
311 ggggaacgagctg tgct acaagg tca tcg tga ccaggga g

TTN (NM_133378)
18171 aaagaacca gctg catttttgaagagattaagtgatca ttc tgta gaa ccaggga agtcc

TUBGCP5 (NM_052903)
1561 cgagtggatcg tgc acggg cacctgt ggg atggcg ccaggga gttcatca

TYSND1 (NM_001040273)
3341 cctggcacttc ctg ga gtgt ggc gtgc ccaggga gggcac

UFSP1 (NM_001015072)
501 ggaggccagcctct gcc tc gctc acttcgga gggc c ccaggga cgcctct

UNC13A (NM_001080421)
7891 aaa tgc aaattctcatacaggg ttg ggaagttg tag ccaggga taagctt

USH2A (NM_206933)
11741 ggccttattctatatttgta gct tgg ata cca ccaggga t

USP35 (NM_020798)
3001 tatg tgctg tttta ccg gcag cgg c ccaggga ggggcccg

VSIG4 (NM_007268)
561 tgacaactggcagcg gtt at ggctt cac ggtgcc ccaggga atgaggatt

VWF (NM_000552)
181 gcaccat tgtc ca gcagc tga gtttc ccaggga ccttgga

WASF3 (NM_006646)
2961 cctccacccttcc tgt tgttccc acgt gggcaata ccaggga cccatggg

WDR60 (NM_018051)
1771 ataa tgttg aaagagaca ttc aaacggaggaaata gag a ccaggga agtg

WIPI2 (NM_016003)
661 aacaacgacaac tgc tac ttggc gta c ccaggga gcgcga

XAF1 (NM_199139)
1721 caat tgcc acaaaaagaataaaatacc tag gaatacag cta a ccaggga g

XIRP2 (NM_152381)
61 aaatgggaatct tgt gattatcagagaa gtga gtg tcat c ccaggga cag

XPNPEP2 (NM_003399)
1941 tc cgtc tcgaagatgtggctctcgtg gta gaagcaaagaccaag tac ccaggga gctacc

XRCC3 (NM_005432)
81 caggagga gcc tcaggagcc ggactgccgtt ggcc aaccgagtcc ccaggga gacactta

ZBTB48 (NM_005341)
581 caagga gccg gcaggcttggaagaagaggaagtttcga ggact ctgggtct agtcc ccaggga

ZFP91 (NM_053023)
3751 tcatttctgaaagag gct tactttataccaactag tgtc agcatttgg gatg ccaggga a

ZIC4 (NM_032153)
781 aaccacatttgctt ctg gga gga gtg tcc gcg ccaggga a

ZNF19 (NM_006961)
541 aaga tgttg agaccaacattgaca gtgagt ccac attaat ccaggga att

ZNF34 (NM_030580)
481 tcacaggagacat ttg gtga gga aga tcc ccaggga tctg

ZNF295 (NM_020727)
1201 c tgtc tattcaccttccatagattt gaa atc ttc ccaggga tcatcttcg

ZNF398 (NM_020781)
4101 cacacttagaaaacatc tgct ccatgac att cac aat a ccaggga ccatg

ZNF452 (NM_052923)
721 gatcaggaatcagccctа cgt aggaatt tgt ctt ata ccaggga actctc

ZNF565 (NM_001042474)
241 ag gtc aaata gttct aaaagcc atggc ccaggga ctggtg

ZNF609 (NM_015042)
4941 ggggcaggg gtt ccaacacc agc acagg gct cc ccaggga

Target site sequences of each mRNA and their first nucleotide numbers are followed by a gene symbol and an accession number. 7-nt downstream and 3- to 5-nt upstream sequences that are complementary to the acceptor-stem and

anticodon-stem domain sequences, respectively, of the 5′-half-tRNA^{Glu}, and a T-arm-like structure sequence in between are shown in red and green, respectively.
