# Supplementary material for: Modulation of Gene Expression by Human Cytosolic tRNase ZL through 5′-Half-tRNA
Source: PLoS One. 2009 Jun 15;4(6):e5908. doi: 10.1371/journal.pone.0005908 (PMC2691602; doi:10.1371/journal.pone.0005908)
Supplement: Table S3 — PCR primers and probes for endogenous mRNA analyses. (0.05 MB PDF) [file pone.0005908.s011.pdf]

Table S3. PCR Primers and Probes for Endogenous mRNA Analyses

gene	forward primer	reverse primer	probe
non-real-time PCR			
PPM1F	5′-ACCAGATGTTTCTCAGGAAAG-3′	5′-GAAGACCACCATGACCGTGAT-3′	
DYNC1H1	5′-CTGCTGAACAAACAAGTCAAG-3′	5′-ATGCCTTCAGCAGGACACGCC-3′	
HDAC4	5′-TGTCTCTGTCTTGAAGCTCAG-3′	5′-TCGTGTCAACTGAATTCGGCA-3′	
FOXP2	5′-CTAAAGAACGCGAACGTCTTC-3′	5′-TGTCAGATGACTCCATGATAG-3′	
28S rRNA	5′-GTTCACCCACTAATAGGGAACGTGA-3′	5′-GATTCTGACTTAGAGGCGTTCAGT-3′	
real-time PCR			
PPM1F	5′-GAAGAGGAGGAGGACGATGA-3′	5′-AAACTCTGTGCCAGGCTTTG-3′	5′-GCTGGATG-3′
DYNC1H1	5′-AGTTGGTGGAATGTGGGTTG-3′	5′-TGATTGATCTGGGTGATCTGA-3′	5′-CTCCAGCT-3′
HDAC4	5′-CACCAACGATTGTGTCTTTCC-3′	5′-AGCCCCTGGGCTTCTTTA-3′	5′-AGGAGCTG-3′
28S rRNA	5′-TTACCCTACTGATGATGTGTTGTTG-3′	5′-CCTGCGGTTCCTCTCGTA-3′	5′-TCCTGCTC-3′
